# Decentralising healthcare for diabetes and hypertension from secondary to primary level in a humanitarian setting in Kurdistan, Iraq: a qualitative study

**DOI:** 10.1186/s12913-025-12571-6

**Published:** 2025-04-15

**Authors:** Éimhín Ansbro, Benjamin Schmid, Ruth Willis, Karwan M-Amen, Kazhan Mahmood, Idrees Abdulkareem, Signe Frederiksen, Jytte Roswall, Sigiriya Aebischer Perone, Bayard Roberts, Karl Blanchet, Nazar Shabila, Pablo Perel

**Affiliations:** 1 Department of Non-communicable Disease Epidemiology, LSHTM, London, UK; 2 Department of Health Services Research and Policy, LSHTM, London, UK; 3 College of Nursing, Hawler Medical University, Erbil, Iraq; 4 International Committee of the Red Cross, Geneva, Switzerland; 5 Danish Red Cross, Copenhagen, Denmark; 6 Geneva Centre of Humanitarian Studies, University of Geneva, Geneva, Switzerland; 7 College of Health Sciences, Catholic University in Erbil, Erbil, Iraq

**Keywords:** Non-communicable disease, Decentralisation, Diabetes, Hypertension, Humanitarian, Conflict, Refugee, Internally displaced person, Iraq, Kurdistan, Primary care, Patient perspective, Qualitative

## Abstract

**Background:**

Experts suggest that Non-Communicable Disease (NCD) care is best delivered at the primary level, including in humanitarian crisis settings. In many crisis-affected countries, NCD care is predominantly delivered by specialists at secondary care level, and there is limited evidence on decentralising NCD care in such settings. We aimed to explore health actor and patient experiences of decentralising diabetes and hypertension (DM/HTN) care from a hospital to primary care clinics in the humanitarian setting of Duhok, Kurdistan Region of Iraq.

**Methods and results:**

We conducted a qualitative study including 30 semi-structured interviews with a purposive sample of patients (*n* = 16), healthcare providers (*n* = 7), and key stakeholders (*n* = 7) involved in the decentralisation project. Guided by a conceptual framework, data were analysed thematically using deductive and inductive approaches. The decentralisation project achieved its stated goals of (a) increasing patients’ access to DM/HTN care, by reducing cost and distance, and (b) decreasing workload at secondary care level. The approach appeared acceptable from patient, provider and stakeholder perspectives. Key health system inputs were put in place to support the decentralisation project, including medicines, equipment and health workforce training, but gaps remained. While access and quality seemed to improve, integration, continuity and sustainability were more challenging to achieve. Key systemic challenges to sustainability included a lack of health financing, and weak national supply chains and information systems. Patients’ trust in the service was important and was closely linked to having access to a continuous supply of trusted medications.

**Conclusions:**

While it is possible to decentralise diabetes and hypertension care from secondary to primary level in a humanitarian setting, multiple contextual factors must be considered, including supply chain strengthening and adaptation to existing workforce capacity. Our study findings may inform other actors exploring the decentralisation of NCD care elsewhere in Iraq and in other humanitarian settings.

**Supplementary Information:**

The online version contains supplementary material available at 10.1186/s12913-025-12571-6.

## Background

Decades of armed conflict, sanctions, sectarian violence, and political instability have severely hampered the capacity of the public health system in Iraq to deliver consistent, high-quality health care [1]. Despite improvements in healthcare access and coverage over the past two decades, major obstacles remain. These relate to the limited financing, governance, information technology systems and health workforce capacity within the healthcare system [2–4]. The system has been further challenged by large population displacements following decades of conflict and disasters. In 2021, there were 1.2 million internally displaced persons (IDPs) and 250,000 Syrian refugees in Iraq, mostly located in the semi-autonomous Kurdistan region (KRI) [5–7]. The global COVID-19 pandemic has placed additional strain on the health system, exacerbating existing service disruption [8].

NCDs caused an estimated 67% of all deaths in Iraq in 2019, mainly related to cardiovascular disease (39%), cancer (9%), and diabetes (6%) [9]. In 2022, almost half of Iraqi adults aged 30–79 had hypertension [9]. Displaced Iraqi and Syrian refugee populations also bear a high burden of NCDs [10]. NCDs have been included in the Iraqi national care package since 2009, and a national NCD strategy, clinical guidelines and essential NCD medicines and equipment lists are all available [11, 12]. However, as in many low-and-middle-income countries (LMICs), despite existing policies aimed at strengthening primary level NCD care, in practice, it remains focused at higher health system levels (i.e. secondary and tertiary hospitals), which limits access and coverage [13, 14].

Little is known about alternative NCD care models specifically targeting displaced populations in Iraq [14]. IDPs and refugees can access the public sector, and international and non-governmental organisations support NCD care for camp-based populations [14]. People living with NCDs also choose to access private care when they can afford it [7, 14]. However, a recent review confirmed that displaced populations’ access to NCD care in Iraq has varied by region and over time, and access is increasingly hampered by economic challenges and stricter work, camp and refugee policies [15]. Decentralisation of health services, that is, moving policy, administrative or budgetary control from central to regional or municipal levels, has been shown to improve access to health services and accountability to local populations [16, 17]. WHO and others have increasingly called for NCDs to be integrated into essential primary health care packages that are accessible to all - including IDPs and refugees - in the drive towards universal health coverage [18–20]. Integrating NCD care into primary care may improve person-centredness, allowing for more holistic, continuous, and multi-disciplinary management of multiple conditions, including, for example, rehabilitation and mental health and psychosocial support [21, 22]. It may also support more efficient use of scarce resources and reduce out-of-pocket spending for patients and their families [21]. Studies on integrated NCD care, mostly from high income settings, have shown both a reduction in the adverse patient outcomes and experiences that may result from care fragmentation, and an increase in service user satisfaction, perceived quality of care, and access to services [21]. Indeed, recent humanitarian operational guidance and a review of global experts’ perspectives suggest that NCD care models should focus on the primary healthcare level in crisis-affected settings and that humanitarian actors should engage in health system strengthening [23–25].

However, there is a major gap in the literature and in operational guidance about *how* to decentralise and integrate NCD care into primary healthcare in humanitarian settings [21]. Since practical examples are limited, humanitarian actors can look to HIV care delivery for guidance [23, 26]. Decentralisation is a key tenet of differentiated HIV care delivery strategies, which conceptualise it as moving care of people living with well-controlled HIV to lower health system levels (“downward referral”) to facilitate universal access to HIV treatment [27–30]. Differentiated HIV care delivery combines decentralisation with task sharing of care to non-physician health workers, strengthening community level- and self-care and integrating HIV care with other primary-level services [31–33]. Lessons learned on improving access and retention in care through decentralisation are salient for other chronic conditions, as LMIC health systems adapt to face the growing NCD burden as well as growing displacement crises [34–36].

**Fig. 1:**
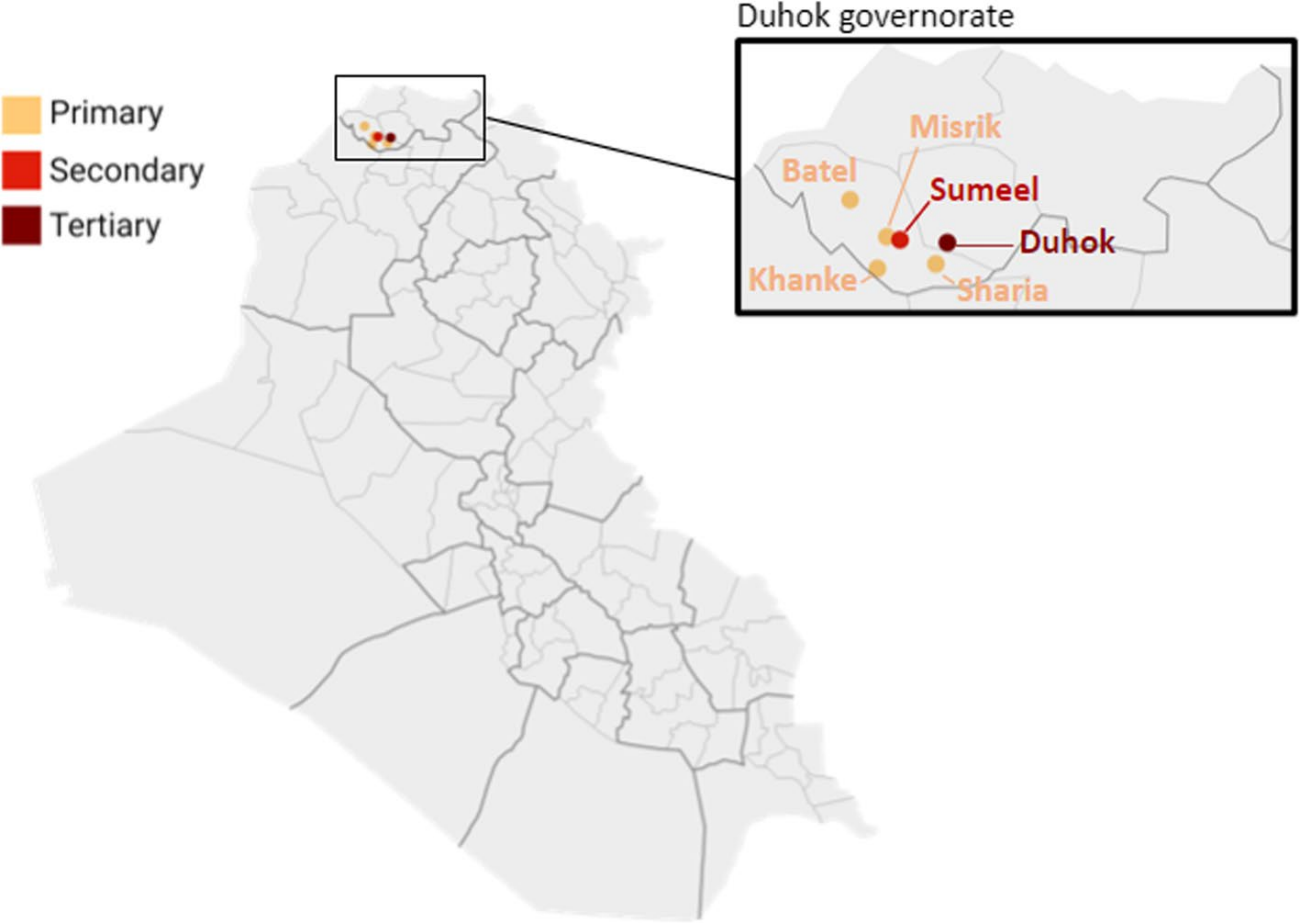
Map of Iraq highlighting the locations of the primary health facilities selected for the decentralisation project [37]

Here, we document the experiences of the International Committee of the Red Cross (ICRC) and Summel Health District Directorate and the Preventive Department of Duhok Department of Health (DoH), who collaborated on a project to strengthen primary-level NCD care in Summel district, Duhok, KRI from 2018 to 2021. The project focused on Summel due to the large number of IDPs residing there and the care gap identified by the DoH. The project sought to decentralise diabetes and hypertension (DM/HTN) services from the secondary care level at Gulan hospital to four primary health care centres (PHCCs) in the villages of Sharia, Khanke, Misrik, and Batel (Fig. 1).

Using a conceptual framework for models of care for NCDs in humanitarian settings, this study aimed to explore the experience of patients, healthcare providers and project stakeholders of decentralising care for people with DM/HTN in the humanitarian setting of Summel district in Duhok, KRI.

## Methods

### Study context

#### Existing NCD services before decentralisation

Duhok governorate is in the north of the autonomous KRI, bordering Syria and Turkey. It has a population of over 1.2 million and hosts around 600,000 forcibly displaced people, including around 498,000 IDPs and 50,000 refugees. Summel, one of seven districts in Duhok governorate, hosts large numbers of IDPs, most (70%) of whom live interspersed within the host community, while the remainder lives in 16 camps. Most refugees in the district reside within four camps (internal ICRC data). Public facilities in the KRI provide NCD care for both displaced populations and the host community. Before decentralisation, DM/HTN care (including diagnosis, consultations, monitoring and medication dispensing) was provided by specialists at each district hospital centre. In Summel district, this was Gulan public secondary hospital (Fig. 1). Gulan hospital issued NCD patients with a chronic disease booklet, which included their diagnosis and medication list. The booklet gave patients access to free-of-charge treatment and medicines, following a small administrative registration fee. It was renewed annually for refugees and IDPs and every two years for the local population. Patients with complex care needs were referred or self-referred to Azadi hospital, the public tertiary referral hospital in Duhok city (Fig. 1). Prior to the decentralisation project, patients did not receive formal NCD care or related medications at the ten PHCCs in Summel district. Community-level NCD support, prevention or sensitization activities were minimal. Private sector providers played a key role and were often preferred by patients. Their reasons included the better availability of medicines (generic and brand names) and the more consistent presence of (specialised) healthcare professionals than in public facilities [14]. These differences were, in part, due to the public health sector’s historic underfunding as well as gaps in regulation of the private sector. Patients also purchased NCD medicines directly from private pharmacies. Despite this preference, private sector care was often unaffordable, and the primary care system played a critical role in ensuring equitable access to care [14].

#### Decentralisation of diabetes and hypertension care in Duhok

Following the launch of the decentralisation project, patients with DM/HTN who previously attended Gulan hospital were advised to attend their local PHCC, where DM/HTN consultations, medicines and laboratory tests (including HbA1c testing) had been introduced. Consultations consisted of monitoring disease control, prescribing and dispensing free-of-charge medications (including human insulin, oral hypoglycaemics and antihypertensive medications) and providing limited healthy living advice. Staff were trained to adjust medications and to refer to Gulan hospital if good control of the NCD condition was not achieved, or in the case of complications. NCD management was based on the DoH guidelines and NCDs were integrated into the Iraqi Basic Health Service Package in 2009 [38].

New DM/HTN diagnoses were made through targeted screening at PHCCs, and these patients were referred to Gulan hospital for formal diagnosis, registration as an NCD patient, and creation of an NCD booklet. The PHCC neither directly provided nor had clear referral pathways for mental health, physiotherapy or palliative care services, for the screening or management of DM/HTN complications, or for related emergencies, such as acute heart attacks or strokes. The project underwent a temporary period of recentralisation during COVID-19-related restrictions (March-May 2020), when NCD services were transferred back to Gulan hospital. Once restrictions were lifted, NCD care was successfully resumed at the PHCCs (internal ICRC data).

The ICRC’s role was to support healthcare provider training, conduct joint quality assurance visits with the DoH, and supply an agreed list of DM/HTN medications and equipment to the PHCCs and Gulan hospital for the duration of the project. The project also involved the provision of new printed information leaflets, which contained information about DM/HTN signs and symptoms, medications and prevention.

### Study design and approach

We undertook an exploratory case study of the DM/HTN decentralisation project, implemented by the Duhok DoH and the ICRC in Summel district, Duhok. The study approach consisted of qualitative semi-structured interviews with patients living with DM/HTN, healthcare service providers, and stakeholders who had been involved in project implementation.

### Study partnership

The study was conducted as part of the Partnering for Change initiative that included the ICRC, Danish Red Cross (DRC), Novo Nordisk, and the London School of Hygiene and Tropical Medicine (LSHTM) as the global academic partner [39]. The research component of the partnership included global-level studies and two country case studies, in Iraq and Lebanon [23, 26]. The Iraq case study was conducted jointly by a research team from Hawler Medical University (HMU) and LSHTM, in partnership with the ICRC; Duhok Directorate General of Health, Department of Planning - Scientific Research Division; Iraqi Red Crescent Society; and the DRC. At the time of the study, both ICRC and DRC provided care for IDPs and the host population in the KRI.

### Conceptual framework

**Fig. 2:**
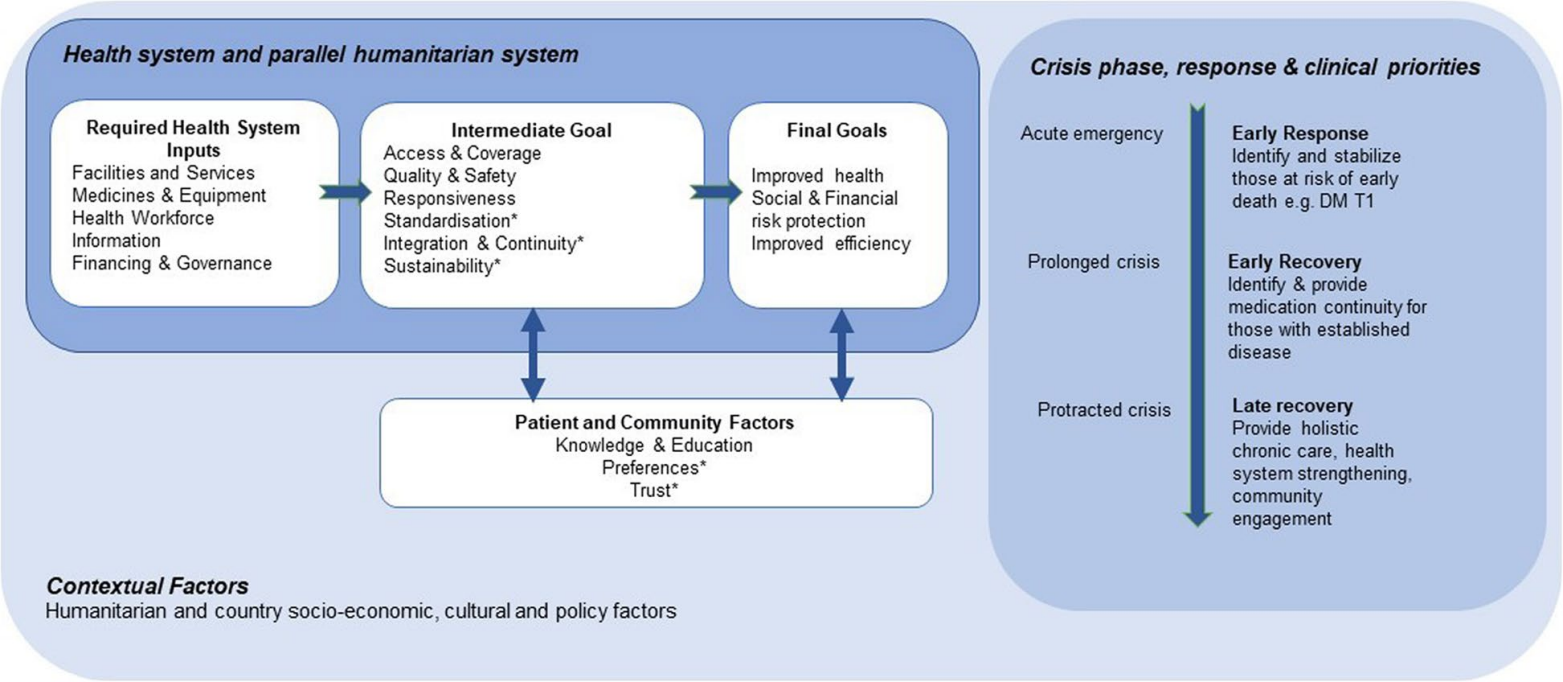
Conceptual framework for analysis of models of care for diabetes and hypertension in humanitarian settings [23]

To guide the exploration of decentralising NCD care in Duhok, the authors used a conceptual framework for high-quality NCD care in humanitarian settings (Fig. 2) that they had previously developed [23]. The framework drew on the World Health Organization (WHO) health system building blocks framework, on prior work done on delivering NCD and mental health and psychosocial support in humanitarian crises, and on the quality-of-care literature [40–43]. The framework helped to operationalise the concept of a model of care and guided the development of study instruments and data analysis and inform policy recommendations. Its components were defined in detail in our related paper and in Supplementary file 1 [23].

### Participant selection and recruitment

Semi-structured interviews were conducted with patients, providers, and stakeholders who had experience with the decentralisation project. Patients living with DM/HTN were purposively sampled from Sharia and Misrik PHCCs, the two largest PHCCs supported by ICRC, for pragmatic reasons. PHCC management staff provided a patient master list, including contact information, and research staff selected a purposive sample of patients to reflect the broader cohort in terms of sex, age, diagnosis and status (IDP, vulnerable host community). The research team (KA, KM) then contacted potential participants by telephone to invite them to participate in the study. We intended to interview people with DM/HTM who were living in IDP and refugee camps and not attending the relevant PHCCs but could not do so due to COVID-19-related travel restrictions. Refugees were, therefore, not included among recruited participants as they lived in camps that were outside the catchment areas of the four ICRC-supported PHCCs.

Healthcare providers from all four of the included PHCCs were purposively selected, based on their involvement in the decentralisation project and on guidance from senior facility staff and study partners. Stakeholders were purposively selected to include a range of people involved with the project, based on a shortlist of potential informants provided by study partners. Both groups were contacted by telephone by KA to invite them to participate in the study and to arrange face-to-face or telephone/online interviews. Patient interview participants were provided with phone credit to compensate them for their time.

### Data collection

Three individual interview topic guides were developed by EA and RW for each of the sub-groups (patients, providers, stakeholders), based on the conceptual framework (available in Supplementary file 2). These topic guides were used in more than one humanitarian setting to explore models of NCD care delivery and were built around the themes of patient pathways, integration and continuity of care, and person-centredness of care. They were adapted to the context and based on pilot interviews undertaken by KA and EA, with further iterative adaptation by the study team as the interviews progressed.

Due to COVID-19-related restrictions during the data collection period, semi-structured interviews were conducted with all patients by telephone, by one female (KM, MPH) and one male (KA, PhD) KMU-based public-health researcher from Kurdistan, in Kurdish. Each was experienced in qualitative research and had been trained on the study objectives, protocol and ethics by the LSHTM team. Interviews with PHCC providers were conducted in Kurdish in person at the facility where they worked by KA. These took place during a period of relaxed travel restrictions, taking appropriate COVID-19 infection control precautions. Other provider and stakeholder interviews were conducted remotely via Zoom in English by EA (female LSHTM-based public health researcher and clinician, experienced in qualitative and implementation research in LMICs, PhD). Three of these interviews were translated from Kurdish in real-time by KA, as required by the language skills and/or choice of the informant.

The interviewers had no prior relationship with the interviewees. Interviewers explained the study objectives, their professional background and their roles in the study. The interviews were conducted between March and July 2021 and lasted approximately one hour. A contact summary sheet with field notes was generated after each interview. All interviews were audio-recorded and no repeat interviews took place. The number of provider and stakeholder interviews was limited by time and logistic constraints. However, the research team felt that theoretical saturation was achieved in relation to the key areas of interest.

### Data management and analysis

During data generation, regular reflective debriefs were held between the HMU and LSHTM teams. Kurdish language interviews were transcribed and translated verbatim by KA and KM. English language interviews were transcribed by EA, RW and BS. Transcripts were not returned to participants. De-identified transcripts were imported into QSR NVivo for Windows^®^ for analysis.

Our epistemological approach was critical realist. An iterative content analysis approach was used, following Braun and Clarke, combining inductive and deductive thematic analysis methods [44]. The trustworthiness of our analysis was enhanced by following these four steps: first, deductive analysis coding to the key elements of the conceptual framework (Fig. 2) (EA, RW, BS); second, inductive analysis seeking to elicit new themes or unexpected findings (EA, RW, BS), which were then examined for robustness in relation to the research question and to existing literature, and incorporated into the coding framework if found to be robust (Supplementary file 3); third, findings were triangulated across sub-groups (patients, providers, stakeholders); fourth, emerging findings were reflexively examined to ensure against the insertion of preconceived assumptions. Throughout the analysis, negative cases or exceptions were examined to test emerging themes and explore why these cases were different. Core codes were developed, applying constant comparative analyses toward categories. Findings were discussed at an online validation workshop with key ICRC and HMU study team members, who were grounded in the study context. We report our findings according to the COREQ checklist [45].

## Results

We conducted 30 interviews in total, consisting of sixteen patients (P), seven providers (PR), and seven stakeholders (KS). The patients’ characteristics are summarised in Table 1. There were no refusals to participate. Most patient participants were male (*n* = 12), despite strategies to support and increase the participation of women. Patients had lived with DM/HTN for over five years, except for one interviewee who was diagnosed in the preceding year. The longest disease durations were 20 and 30 years, involving diagnoses of DM in childhood.

Providers included pharmacy managers (*n* = 3), clinic managers (*n* = 1), and decentralisation project staff working at the facility level (*n* = 3). One provider held a joint role as a pharmacy manager and nurse. Providers from three PHCCs and Gulan hospital were interviewed, as the research team could not establish contact with the fourth facility (Khanke) despite repeated attempts. All interviewed providers, except one, were male, which reflected the DoH’s assignment of health professionals to the decentralisation project, rather than the general gender distribution of providers at the facilities (roughly 40% female).

Of the seven interviewed stakeholders, most were in project management or coordination positions (*n* = 5), while two were implementers at the community level, not directly involved in the decentralisation project.

**Table 1:** Characteristics of interviewed patients (*n* = 16)

Characteristic		Total number
Gender	Male	12
	Female	4
Status	Host community	4
	IDP	12
Diagnosis	DM	9
	DM&HTN	7
	HTN	0
Time since diagnosis	Mean (range)	10 (1–30) years; *n* = 13
Location	Sharia	8
	Misrik	8
Age	Mean (range)	50 (30–68) years

### Decentralisation of DM/ HTN care in Duhok

We describe the implementation of the Duhok DoH/ ICRC decentralisation project and lessons learned, guided by our conceptual framework (Fig. 2). No data on the *final goals* domain of the framework are presented, given that the qualitative methodology did not allow us to examine project impact.

### Context, crisis phase, and broader system

Multiple stakeholders noted that the project was implemented in the context of a difficult public health system financing landscape, with the Duhok health system dependent on limited central funding from Baghdad amid an economic crisis. Some noted the deterioration in the health system from its heights in the 1980s, due to repeated outbreaks of conflict and instability. They also noted dwindling donor funding for Iraq’s protracted crisis.

### Health system inputs required for the decentralisation project

Key health system inputs required strengthening to support decentralisation of NCD care to the PHC level. Here we report against the WHO health system building blocks (which were included in the conceptual framework).

#### Financing, leadership and governance

Strengthening primary level NCD care had already been included by the Duhok DoH in their health care policy prior to the decentralisation project. However, according to some stakeholders, the DoH lacked the autonomy to implement its policies. This was, in part, because the DoH was reliant on stretched national Iraqi Ministry of Health financing and poorly functioning supply chains. This meant that they were partly dependent on international non-governmental organisation (NGO) funding and, therefore, tied by the decisions and diverse programming preferences of NGOs:

*“Each [NGO]*,* has their [own] proposal and funds…and also*,* we have problems and we need anyone [to] help us. So*,* we can’t introduce [our own proposals].” Stakeholder 01*.

A key theme generated inductively from the data was the role that international humanitarian actors may play in relation to national health systems strengthening and their flexibility or otherwise to adapt to the context. Stakeholders described humanitarian actors’ autonomy being limited by donors’ short-term funding cycles and their increasingly prescriptive role:

*“[…] one of the problems [is that] donors […] are getting more and more involved on what we’re doing. So […] donors who do not really have the sense of what’s going on in the field are dictating us how to use the money.” Stakeholder 06*.

When probed for lessons learned around the decentralisation project, multiple stakeholders emphasised the need for strong coordination and communication between the implementing institutions. Local stakeholders recommended paying incentives to healthcare staff in future, to encourage their involvement in the project and compensate for undertaking additional tasks. However, other stakeholders discouraged this, since incentives introduced on a project-by-project basis were not likely to be sustainable.

#### Services, medicines and equipment

After decentralisation, all patients with DM/HTN, irrespective of clinical severity, were advised to attend their local PHCC for ongoing care. If visiting Gulan hospital unprompted, patients were advised to seek care from their catchment PHCC and were not provided with medicines or services.

*“Simply all those patients [at the hospital] were notified not to come back to Gulan unless they were referred […]. This went on gradually until we saw a big decline in the number of people visiting the central hospital and gradual increase in the numbers of people visiting [the PHCC].”* Stakeholder 04.

Patients could visit the PHCC at any time during opening hours without an appointment for medical consultation, but were required to adhere to pre-set appointments for monthly medication refills. Disease monitoring and laboratory testing could be performed during their consultation visit, although this was not systematically done, and some patients reported prompting staff to conduct monitoring:

*“Even for the blood test*,* we have to ask them to do the test. Otherwise*,* they don’t do it regularly.”* Patient 15.

Providers reported giving limited lifestyle information to patients and specifically requested additional training to strengthen this area of care. The attendance rates at the PHCC level closely mirrored changes to the provision and availability of medicines at the PHCC, the Gulan hospital and other local providers, described in more detail below.

Medicine availability and quality featured as prominent themes in all interviews. The decentralisation project was reportedly planned and built around medicines supply, rather than being patient- or service-centred, and key stakeholders emphasised that medicines’ supply underpinned the project:

*“[To implement this project the] first and [most] important thing is availability of the medicine… If the medicine is not available*,* we cannot do anything and if we do anything it is not important.”* Stakeholder 01.

ICRC and the DoH negotiated a list of ten medicines, based on the WHO Essential Medicines List (EML) and Package of Essential Noncommunicable Diseases Interventions, that the ICRC procured internationally and initially delivered to the facilities via a parallel supply chain. This medicines list did not align with the medicines that doctors were used to prescribing and that patients were used to receiving, and the DoH chose to supplement it with medicines more typically prescribed by their doctors (e.g., analogue insulins, which were not included at the time of the study but have since been added to the WHO EML). The DoH supply chain prioritised supply to Gulan hospital, and stakeholders and providers described regular stockouts of NCD medications occurring in PHCCs towards the end of each month:

*“We did not face any problems with the medications that are supplied by the ICRC*,* but [those] not supplied by the organisation*,* we have shortage of them. Gulan has the medication*,* but they don’t give it to us*,* and this created a problem for us.”* Provider 04.

Various stakeholders described systemic issues which contributed to medication and equipment stock-outs. These included the national supply chain receiving insufficient funding and operating a push system based on central medicines availability and outdated population data (not accounting for IDPs’ movement) rather than on local needs. Due to these challenges, supply did not match consumption. In addition, consumption monitoring and inventory management were reportedly limited within the public service and several respondents suggested a need for supply chain forecasting training for PHCC staff.

Many patients reported purchasing medications from the private sector, especially when their prescribed– or preferred– medication was unavailable from the PHCC, and many described this as unaffordable. Some patients would attend private practitioners in addition to the public facilities (sometimes prompted by public sector doctors).

*“I usually go to the centre but sometimes I [have to] buy the medication in the pharmacy [when it is unavailable in the centre] It is expensive*,* but I have to.” Patient 04*.

Care for diabetes and hypertension was decentralised but care for comorbidities, such as cardiac disease, remained at Gulan hospital level. Therefore, some patients with comorbidities had to attend both their local PHCC and Gulan hospital to obtain their full list of medicines.

#### Health workforce

Patients received care from a team of non-specialist doctors, nurses, pharmacists and laboratory staff at each PHCC, although the staffing numbers varied widely across centres. Senior PHCC staff and stakeholders noted that the PHCC level providers were understaffed, overworked, and were expected to multi-task, both before and after decentralisation. For example, it was noted that staff were too busy to enter paper-based data into the electronic database specifically created for the project. Despite this workload and that their salaries were often unpaid, as reported by one DoH stakeholder, it appeared from staff accounts that many staff were highly committed and they endeavoured to provide good quality patient care, for example:

“*We do our best to avoid crowdedness in the [PHCC] and to make patients happy with [our] services.*” Provider 05.

By contrast, providers at Gulan hospital were less busy post-decentralisation, having gone from seeing approximately 120–160 patients per day to 60 patients per day. The additional time available for each patient consultation was seen as likely improving the quality of care.

Stakeholders involved in decentralisation reported that the DoH prepared a training package of fourteen modules for PHCC and Gulan hospital staff, delivered by DoH specialists, and supported by ICRC. The DoH and ICRC stakeholders reported conducting monthly supervision visits to check prescribing patterns, pharmacy management and reporting, patient follow-up and staff concerns. The training initially targeted doctors but was adapted to focus on nurses and ancillary staff, since there was much lower turnover of these staff cadres compared to doctors. The latter worked at multiple facilities and were required to undertake one-year placements in rural PHCCs before moving on to further training.

*“So that is why we decided to focus on the health staff that are the PHCs– the health staff the nurses*,* the lab technicians and the pharmacist assistant. These people […] don’t rotate*,* they are stationary there and that is why during the last year of the programme we focused our capacity building [them].*” Stakeholder 04.

The training covered administration (registering patients, and appointment and referral systems), managing patient interactions, and, for those providing consultations, adjustment of medications, including anti-hypertensives and insulin. Refocusing the training to target more permanent members of staff was a key lesson learned during implementation as it helped ensure that capacity building was sustained.

Most interviewed providers felt that they received insufficient DM/HTN training. Several noted that additional supervision and training, particularly on diet, would be valued:

*“When the patients come here*,* they should be made aware [to] avoid eating salty and fatty foods*,* as they are dangerous for [their] health […]. We don’t have that knowledge*,* […] we are poorly educated about nutritional health.”* Provider 05.

Patients and providers further recommended increasing the number of staff and having specialists present at the PHCCs to improve care.

Some stakeholders noted that medical training in Iraq contained limited focus on NCDs and primary care and this was perceived as a barrier to decentralising NCD care more broadly in Iraq. Nurses were considered by one stakeholder as a potentially untapped resource for the decentralisation project and the wider Iraqi health system, if provided with substantial additional training, ideally via the national medical education system:

*“It is important to ensure continuous training for health professionals*,* not just when ICRC is there to train the people. Or to integrate that in the curriculum of the university […]; to get involved in the education of new professionals.”* Stakeholder 03.

#### Information systems and data sharing

The paper-based patient file system that existed prior to decentralisation was maintained. Before decentralisation, there was a lack of formal communication and information sharing between different health system levels and patients continued to hand-carry their information via their patient booklet. According to one stakeholder, this was an important barrier to the decentralisation process:

*“ [the] lines of communication between the central clinic and the periphery were not established and we did not have the budget or the capacity to establish a new form of communication.” Stakeholder 04*.

Some stakeholders described the initiation of a social media group for clinical staff to communicate between the four PHCCs and Gulan hospital to act as a proxy referral system. However, neither the ICRC nor the DoH were willing to take ownership of maintaining this, while the DoH planned to invest in a new computer network to support referrals. However, they did not have the funds to do so at the time of the study.

### Patient and community factors

Most respondents agreed that the patients involved in the decentralisation project, and the Iraqi population in general, preferred private, specialist care and chose to avail of it when they could afford it. Patients seemed to have less trust in public sector services and medications compared to the private sector. Providers also felt that patients preferred to attend hospitals rather than the public primary care system, which they perceived as “*weak*”. According to key stakeholders, patients’ trust in PHCC services was more closely linked to having a consistent supply of medicines, rather than to the presence of the doctor.

“*[Consistent supply of medicines] is one of the most important things*,* it’s even more important than the presence of a doctor. A lot of cases we faced absence of a doctor until a replacement was recruited*,* we did not notice any drop in the attendance rate. But once a medicine or a test is missing*, *then the number will go crazy.*” Stakeholder 04.

Patients’ trust in PHCCs was also shaped by the medications’ packaging. Project medicines were dispensed in ‘unsealed’ small bags, decanted from large containers. Respondents reported that this had previously been standard practice in the public healthcare system, but that boxed medications were now used to address historical challenges around substandard and counterfeit medications. Patient, provider and stakeholder respondents all noted that unsealed medication was less acceptable to patients, for example:

*“Sometimes patients don’t trust the unsealed medications and they…rejected [it]. They go to [private] pharmacies or other places because they don’t want to take the unsealed medication.”* Provider 02.

Patient accounts reinforced this point. They prioritised the presence of high-quality medicines at the PHCC level. To them this meant that medicines should be dispensed in sealed packaging, have good patient-perceived efficacy, and be manufactured by a ‘*good’* producer.

According to most participants, patients’ income levels were highly influential on their acceptance of decentralisation of their NCD care to PHCC level, essentially because they could not afford any alternative:

***“****They will give you unsealed medication and [most of the] people here are poor and take the medication.”* Provider 05.

### Intermediate goals

#### Access and coverage

Key stakeholders reported that the project had achieved one of its key aims of improving patients’ access to NCD care. It clearly reduced distances and improved convenience for most patients by bringing the services closer to home. Most patients described access as “*easy*”, “*convenient*” and “*free*”. (Patients 04, 08). This contrasted with Gulan hospital, which *“[required] money*,* time and transportations*” (Provider 03). Decentralisation was particularly beneficial for older and more financially constrained patients:

“*The patients like to get their treatment here [at the PHCC] because they are IDPs and older men and women. They cannot go to Duhok every month.”* Provider 03.

In terms of equity of access, several providers emphasised that access and treatment at the PHCC were the same for the local population and IDPs. Affordability of DM/HTN services at public facilities was unchanged post-decentralisation, as care continued to be provided free of charge except for a nominal, “*affordable*” co-payment of 500 IQD (around 0.4 USD). Patients’ main affordability concerns were the cost of transport and co-payments for laboratory investigations.

#### Quality and safety

Reducing the workload in Gulan hospital was reportedly the second key aim of the decentralisation project. Several stakeholders suggested that this goal was achieved, reporting a significant drop in patient load which allowed for longer consultation times. One stakeholder interpreted this as potentially improving quality:

*“We were able to decrease the [work]load tremendously in [Gulan] […] so that eventually the access and quality is improved. I would say [this was] the biggest success [this project] achieved.”* Stakeholder 04.

Key stakeholders reported observing improved clinical indicators for selected DM/HTN patients during the routine project monitoring and quality assurance visits to the PHCCs:

*“I think […] the availability of medicines and the capacity building […] contributed to a very accept[able] level of quality of care in the [PHCCs].” Stakeholder 04*.

Most interviewed patients seemed satisfied with the care provided at the PHCC level, commenting that it was *“better and nice”* (Patient 01) compared to going to Gulan hospital. Patients perceived staff to be doing the best they could with the limited available resources.

*“it’s good*,* I’m satisfied. They don’t have capabilities. The staff are not getting their salary enough and they [still] offer these services”. Patient 08*.

Asked about the quality of NCD care at PHCCs, many patients commented negatively on the lack of specialists and felt PHCC doctors’ knowledge was inferior to hospital-based doctors, for example:

*“A doctor will be in the [PHCC] everyday…he will not do the check up for you*,* he will not do nothing for you. He just writes down the medication on my booklet.”* Patient 12.

#### Responsiveness

There were no references to most of the responsiveness subthemes, such as autonomy, choice and dignity. Other themes, including confidentiality and quality of amenities, were discussed to a limited extent. Most patients complained that the public PHCCs were crowded, and one patient discussed the lack of privacy at all public facilities:

“*In public centres or hospitals*,* the doctor [is] seeing women and men patients all together and you cannot have any privacy.” Patient 12*.

Generally, patients reported the facilities to be “*clean*” and adequately spacious. Despite the reported overcrowding, patients and providers mentioned short waiting times and that patients were treated well, with respect, and equally. Several patients commented that doctors were “*helpful”* and had good familiarity with patients, “*they know me” (Patient 05)* and doctors were “*like a friend”.* There was a sense from some patients that interactions were brief and perfunctory, for example, “*You know how it is*,* they give us our medication and* we go.” (Patient 08). Several providers and one patient offered examples of staff responding to patients’ needs, for example, speaking in Arabic for Arabic-speaking patients.

#### Integration, continuity and standardisation

Continuity across health system levels was a particular challenge. While specific referral criteria and documents were in place to refer from primary back to secondary care, referrals relied upon patients’ hand-carrying referral documents and communicating their clinical history:

*“If a patient visits another hospital*,* private or governmental*,* there is no shared information [and it is up to the] patients to say what they had in the past and are currently receiving”* Provider 03.

Continuity, in terms of longitudinal patient contact, was supported by an appointment system but hampered by a lack of reminder and defaulter tracing systems. Stakeholders suggested that patients perceived a lack of continuity at the PHCC level, in terms of personnel and medicines, which contrasted to their experience at Gulan hospital with staff whom “*they know*” (Stakeholder 01):

*" Sometimes [the patients] don’t like the doctor in the PHCC*,* because [they] just work three days per week. When [the patient] needs their medicine*,* [the doctor] is maybe not available.”* Stakeholder 01.

As this perception was affected by the high turnover and lack of doctors it may have been counteracted– to some degree– by training PHCC nurses to undertake consultations. Patients and providers suggested that improving the appointment system to ensure closer patient follow-up would also support better continuity of care.

There was limited discussion of standardisation of care. Standardisation in prescribing was still an issue, as mentioned above, with providers preferring to prescribe familiar drugs rather than those included on the project’s agreed medicines list. Several stakeholders noted the impact of locally operating NGOs on the project’s sustainability:

*“A lot of times NGOs came in at the end of the year*,* wanting to spend all of their budget. So*,* they go to local market and buy the most expensive antihypertensive and hand it over to people like candy. We were trying to establish something for the long run and those temporary interventions always took us a step back.”* Stakeholder 04.

Stakeholders mentioned the need for improved coordination across NGOs in Duhok, for example using the same patient booklet and medicines list, to promote standardisation and continuity of care.

#### Sustainability

According to our respondents, the ICRC aimed that the decentralisation project would be sustained beyond their involvement. This was reflected in multiple project components, such as the use of existing DoH clinical guidelines and trainers, alignment with the national basic care package, anchoring in institutional DoH and ICRC policies, joint exit strategy planning, purchasing of equipment from the local market, and choice of affordable– rather than “*super new*” (Stakeholder 03) medications. One stakeholder reported a “*long journey*” to developing agreements around these key project components, with sustainability as a “*main goal*” (Stakeholder 04). Another described the DoH as the lead implementer with the ICRC in a supporting role.

There was tension evident within the accounts of the ICRC and their perceived role and ethos as emergency responders in conflict settings versus the system-strengthening needs in Iraq. ICRC’s policy was to build capacity and hand over, ensuring the DoH’s primary role:

*“I think we [tried] a lot [to promote sustainability]. The trainers were not [ICRC] trainers but [DoH’s] trainers […]. It’s not [the ICRC] doing the clinic*,* it’s them. I think [that] kind of involvement is very important.”* Stakeholder 03.

Meanwhile, the DoH stakeholders perceived the project to hinge on the financial support and supply of medications, and thus, on further financial support from ICRC:

*“Of course*,* [the NGOs support] is [only] for [a] short period. What about after two years? You want the DoH [to do it]? Yes*,* the computer [is there] and the data [are] available. But if the pharmacy is empty*,* if the lab [has no equipment]*,* how can I [maintain disease] control and decrease the number of the complications for [NCD patients]?*” Stakeholder 01.

Despite extensive efforts to ensure sustainability of the decentralisation project, multiple stakeholders recounted the challenges that occurred after ICRC’s handover. The main barriers included the DoH’s inability to take over the project’s financing, particularly the supply of medications, and the loss of knowledge due to a turnover of PHCC staff and senior DoH personnel:

“We still we have everything but the number [of patients attending has] come down. Why? Because of [a] shortage of medicines.” Stakeholder 01.

“The [incoming DoH staff] were not aware of the challenges, the long [project] journey. So, we are facing challenge[s] to make the new administration realise [what the DoH achieved] and [what] is needed to maintain those achievements.” Stakeholder 04.

The existing exit strategy was seen as insufficient by some stakeholders, who recommended a more proactive approach to planning the project handover strategy, including securing follow-up funding before it closed.

## Discussion

This qualitative study explored the patient, provider, and stakeholder experiences of a humanitarian actor supporting a public health system to decentralise care for DM/HTN from secondary to primary care level in Summel district, Duhok, KRI. The decentralisation project reportedly achieved its stated goals of increasing patients’ access to DM/HTN care, by reducing cost and distance, and decreasing workload at the secondary care level. The approach appeared acceptable from patient, provider and stakeholder perspectives.

Several key facilitators and barriers to its implementation emerged (Table 2), and questions were raised about the sustainability of the project. ICRC and the DoH collaborated to put many of the key health system inputs in place to support the decentralisation project. Nevertheless, key gaps remained. While the intermediate health system goals of access and quality seemed to improve, it was more challenging to achieve others, such as integration, continuity and sustainability. ICRC financing and support for the consistent supply of medicines was for a limited timespan; staff turnover in clinics and within the DoH limited continuity of care and sustainability of governance; while limited information systems and lack of formal referral pathways hampered both continuity of care and integration between different health system levels.

It is widely acknowledged by WHO and others that most NCD care should be integrated into primary healthcare, with a cohesive approach to prevention, early detection and high quality, holistic management, spanning rehabilitation to palliative care. Literature on NCD interventions from humanitarian and stable LMIC settings highlights the existing gaps in key NCD-related health system inputs, especially since primary care systems in many LMICs are still oriented towards providing acute, episodic care [23, 46]. When designing NCD care models, humanitarian actors have, therefore, focused on establishing these key health system inputs, such as a workforce trained in primary level NCD management, while the intermediate health systems goals, such as quality and continuity, have often been an afterthought [23, 26]. In our study, stakeholders perceived that the decentralisation project improved the quality of NCD care at primary and secondary levels, linking this to quality assurance visits, staff training, and to reduced workload at hospital level. Other authors have found that it is vital to go beyond training primary care staff on clinical NCD guidelines, and to engage more widely in health system strengthening, including improving equipment and medication supply chain and information systems, to achieve high quality NCD care at primary level in humanitarian settings [47].

Among the health system inputs, a continuous supply of medicines was considered the most critical by our study respondents. This influenced patients’ trust in the system and moderated their care-seeking behaviour. ICRC procured the agreed list of medicines internationally based on strict quality standards, but patients did not trust them because they were ‘unsealed’. Historically, unsealed medication was used in the public sector in Iraq and patients associated this with substandard and counterfeit items. Patients’ main recommendation for improving the decentralisation project was the consistent provision of boxed medicines. This example of mistrust in a specific project component is likely intertwined with historical mistrust in the public healthcare sector and a linked preference for private healthcare, described in Iraq and other settings [15, 48–50]. Some providers and stakeholders also perceived that medicines in the public sector were of lower quality than those available in the private sector and described recurring stock-outs at public facilities, which were not involved in the decentralisation project. Patients in our study also seemed to distrust primary care in general and to prefer specialist care, although a national household study on attitudes to primary care in Iraq reported relatively high levels of trust [51]. Trust has been noted as a key component of high-quality healthcare systems more broadly, while some authors argue that it is even more important for NCDs, given the need for continuous care and repeated contact with the healthcare system [50, 52]. Developing trust is a complex issue, and our study shows that understanding local opinions is crucial, since minor changes, such as the use of unsealed medication packaging, may influence patients’ trust and engagement with a project. We suggest that using participatory approaches with patients and providers to design new models of care could help anticipate and resolve some of these issues.

Despite its perceived success and the DoH’s motivation to continue it, the project’s sustainability proved challenging. ICRC had included extensive and deliberate efforts to ensure sustainability, such as supporting the DoH to lead training and supervision visits and creating a detailed handover plan and documents. Despite these efforts, fundamental systemic issues remained after their support was withdrawn. The DoH was dependent on limited national funding and a weak national supply chain, meaning there was a risk of interruption of NCD medication supply to the PHCCs.

Decentralisation successes could be threatened by renewed health system disruption, including outbreaks of conflict or other hazards, as clearly demonstrated by the impact of the COVID-19 pandemic on the project. The WHO Eastern Mediterranean Regional Office has recently produced a regional framework for action on NCDs in Emergencies and is encouraging an “All Hazards” approach to health systems preparedness, response and resilience planning [53]. We note that the public health system in the KRI has already included access for IDPs and refugees. It is essential to build on this example, and to include NCDs in Iraq’s efforts to implement universal health coverage, integrating NCDs into comprehensive primary care, which spans the continuum from prevention to palliative care, while ensuring sustainable financing and including forcibly displaced populations.

A key theme generated inductively from the data was the perceived internal tension between ICRC’s identity as a humanitarian organisation that engages in alleviating immediate suffering, and their health system strengthening role in this project. Thus, this study illustrates the humanitarian-development nexus in action. NCD care in protracted humanitarian settings requires a more development-oriented approach, with a multi-annual strategy and budget, and a system-strengthening mindset. This may require a shift in thinking from humanitarian actors as well as from donors engaged in NCD care in crisis settings, who have both tended to focus on more short-term goals and financing [33].

Much has been written on how the key lessons learned in delivering chronic care for HIV and tuberculosis at the primary level may be adapted to NCD care in LMICs [54–56]. The WHO’s differentiated framework’s recommendations for strengthening HIV care could be used as a blueprint for strengthening primary level NCD care [33]. These recommendations underscore some of the gaps that remained in the Duhok decentralisation model, including clearly defining who can be treated at each health system level and when to refer; strengthening systems that support decentralisation; integration (including referral pathways) and task sharing; strengthening supply chains; and investing in data systems for patient tracking and for project monitoring and evaluation [33, 34, 57]. Interestingly, in this study, the high turnover of doctors prompted task sharing with nurses, and the project’s guidelines and training were adapted to their needs. Nurses were seen by some respondents as an untapped resource, which may warrant further exploration as Iraq seeks to strengthen primary level NCD care more broadly. There is also potential to develop more community-focused models of care in this setting, again learning lessons from the recent experiences of integrating NCD and HIV care, by for example, using community adherence clubs and peer support groups [34].

### Recommendations for implementation, policy and research

We recommend that the KRI and Iraqi governments continue to strengthen the integrated delivery of NCD care at primary level and include NCDs in an All-Hazards approach to health system preparation, response and resilience. We encourage health service delivery practitioners and clinicians to implement high quality primary level NCD programmes, underpinned by evidence-based clinical guidance, regular training and supervision, robust and responsive supply chains and information systems, and sustainable financing. Future care delivery models could build on the task shifting to nurses that was demonstrated here and could explore community-based delivery and patient empowerment models. Implementation research is essential to learn lessons that may improve care and may support opportunities to scale up or translate new care models to other settings. We have outlined additional specific policy recommendations in Table 2.

**Table 2:** Facilitators and barriers to decentralising NCD care in Dohuk, Iraqi Kurdistan, and implementation and policy recommendations

	Facilitators	Barriers	Recommendations for implementation & policy
**Context**,** crisis phase**,** and broader system**	• Humanitarian actors’ financial support and their own NCD agenda, coupled with the pressing health needs of the displaced and host populations acted as a potential catalyst for health system reform and strengthening	• Decades of war weakened the Iraqi public health system • NCD care was historically concentrated at tertiary health system level in Iraq • This was a protracted humanitarian crisis, with donor funding dwindling and humanitarian actor’s remit expiring • COVID-19 pandemic temporarily reversed decentralisation	• Investigate more effective strategies for humanitarian actors’ engagement with governments, development actors and funders, to promote alignment around response and rebuilding activities and support longer term health reform • In keeping with the “health for all” paradigm, NCDs should be integrated into strengthened primary health care within a universal health care approach, and access must be extended to people who are forcibly displaced by humanitarian crises • NCDs should be integrated into an “all hazards” approach to health system preparedness, response and resilience emergency planning
Health System Inputs			
**Financing**,** leadership and governance**	• Strong coordination and communication between the implementing institutions • Intention to support sustainability by casting the DoH as lead organisation, with ICRC in supporting role • Financial support from ICRC for a list of ten core NCD medications • ICRC support to develop training & undertake supervision and quality control visits; however, ownership was retained by DoH	• Insufficient and intermittent funding from central government • DoH finance package and medicines supply did not account for influx of IDPs into Dohuk • National supply chain operated as a “push” system and is not needs-driven; limited drug consumption monitoring systems • Financial constraints meant DoH was partly dependent on other actors & their agendas • Humanitarian actors were constrained by donors’ short term funding cycles and agendas • Turnover of DoH and ICRC staff meant institutional knowledge and “buy-in” was lost	• Actors engaged in providing NCD care in humanitarian settings should consider taking a more development-oriented approach, with a multi-annual strategy and budget, using a system-strengthening mindset • This may involve humanitarian actors working closely with the national health system and/or development actors and funders • Maximise collaboration between health actors involved in NCD care to minimise fragmentation and support continuity of care
**Services**,** medicines and equipment**	• Medicine availability and quality were a key facilitator for project success • Clear clinical and operational guidelines for primary level HT/DM care created, including referral guidelines and pathways back to secondary care	• Mismatch between procurement and delivery of ICRC- and DoH-financed medicines • Mismatch between ICRC-procured medicines & doctors’ prescribing behaviours • Prioritisation of medicine supply to hospitals when stock was low • Regular stockouts of medicines and equipment at PHCCs hampered continuity of care • Partial decentralisation to primary care (e.g., care for HTN/DM but not for comorbidities)	• Use the national supply chain, where possible, rather than setting up parallel systems • Where there are doubts about quality, consider supporting the national system to improve consumption monitoring and quality control processes, where possible • Follow national clinical and operational guidance, where possible, using a rationalised medication list
**Health workforce**	• PHCC staff commitment and effort to provide good quality care • DOH-led training and monthly supervision visits facilitated sustainability and owner • Involvement of and task-shifting to nurses and ancillary staff, including refocusing training on these more permanent staff • Staff respect and kindness cited by patients as facilitator to attendance	• Understaffed, overworked PHCC level health care providers who received salaries only intermittently • High turnover of doctors at PHCC level and frequent gaps in availability • Lack of training on dietary components of NCD care for PHCC providers • Limited focus on NCDs and primary care in medical training in Iraq	• Financial support for the national system • Explore efficient models of care, e.g. promoting self-care, efficient review intervals, decouple consultation from medicine dispensing • Improve HCW training on diet and exercise recomendations • Work with medical training bodies to improve training on NCDs, including for doctors and nurses at primary care level
**Information systems and data sharing**	• Ad-hoc communication platforms set-up for the project • Existence of a patient-held clinical file facilitated some continuity of care between primary and secondary health system levels	• Lack of formal communication and information sharing between health system levels • Patients were responsible for transmitting information between primary and secondary care	• Implement computer network or mobile phone-based referral processes, with due diligence around data security • Ensure two-way flow of information from referring site to referrer and back • Set up key affordable, accessible referral pathways, required for NCD care
**Patient and Community Factors**	• Regular availability of medicines influenced patient attendance and trust at primary care level	• Primary care HCWs were perceived as less good, under pressure but doing their best, compared to secondary level HCWs • ICRC procured medicines dispensed in “unsealed” plastic bags, rather than branded boxes, which diminished trust in the quality of medications • Historic and institutional mistrust in the public sector and patient preference for specialist care	• Strengthened supply chains with consumption monitoring and a “pull” supply chain would help reduce stockouts and resultant distrust in primary level NCD care • Patient education around medicine quality may help improve trust in “unsealed” medications • Participatory approaches to programme design may anticipate and help mitigate issues influencing trust in and attendance at PHCCs
	• Patient cohort income levels and ability to afford care acted as both a facilitator and barrier to attendance at PHCC		• Consider covering transport fees as part of the care package
Intermediate Goals			
**Access and coverage**	• Close proximity and easier access to primary care facilities • Free care at primary and secondary public facilities	• Patients with comorbidities, such as CVD, were required to attend both their local PHCC (for HTN/DM care and medication) and the secondary hospital	• Moving NCD care closer to patients improves physical access and affordability, but must be underpinned with regular access to high quality medications and rationalised investigations, as well as to health workers trained in NCD care
**Quality and safety**	• Project monitoring & quality assurance visits to the PHCCs reportedly improved quality of care • Reduced patient numbers in secondary care increased time doctors spent with individual patients, enhancing quality of care • Patients’ perceptions of facilities were positive (e.g., access, cleanliness, privacy)	• Medication stock outs, high turnover or absent medical staff hampered continuity of care	• Integrate regular quality assurance practices into programming, including supportive supervision and clinical audit • Implement regular refresher training, acknowledging high staff turnover • Included patient-reported outcomes measures as part of quality assurance
**Integration**,** continuity and standardisation**	• Introduction of an appointment system • In response to the high turnover and lack of doctors, the project leadership introduced task sharing to nursing staff, who were more permanent than doctors	• Lack of reminder and defaulter tracing systems • Lack of formal referral pathways, relying instead upon patients to hand-carrying referral documents and communicate their history to clinicians • Lack of standardisation in prescribing practices • Lack of coordination amongst humanitarian actors and other actors’ “temporary” interventions	• Implement appointment, reminder and defaulter tracing systems to facilitate continuity of care • Implement evidence-based guidelines, based on national guidance • Ensure health care workers receive regular refresher training on guidance, supporting standardisation and continuity of care • Humanitarian actors should work closely with national health systems, to strengthen them and support sustainability
**Sustainability**	• Project’s focus on building existing capacities (e.g., DoH clinical guidelines and trainers, aligned with national basic care package) • The establishment of a dedicated exit strategy	• Short-term programme funding threatened sustainability • Lack of financial capacity of DoH to take over project’s financing, particularly the supply of medications • Loss of project knowledge due to turnover of PHCC staff and senior DoH personnel	• Humanitarian actors involved in NCD care should engage with all relevant stakeholders, especially Ministries of Health, UN agencies, Health Cluster members, development actors and funders on the needs, scope and goals of an NCD intervention, aligning with pre-existing health system reform.

### Strengths and limitations

This study is the first to document the experiences of an NCD care decentralisation project in a humanitarian setting from multiple perspectives. It may provide valuable insights relevant to other decentralisation efforts, which were enabled by our use of a conceptual framework for high-quality NCD care in humanitarian settings. Despite the onset of the COVID-19 pandemic and related movement restrictions, we undertook thirty interviews from multiple participant categories, including internally displaced persons and the local host population, and the team felt that theoretical saturation around our key themes was achieved.

However, most interviews were done by phone due to the restrictions, including some stakeholder interviews in English, and this may have hampered rapport-building. Interviewers were selected purposively, based on recommendations from the implementing organisations, and were limited to a subset of PHCCs involved in the project for pragmatic reasons. Given the role of the ICRC in the selection of respondents, there may have been some selection bias in our sampling approach. Despite efforts to assure participants of their anonymity, social desirability bias may have shaped interviewees’ answers, particularly for patient respondents. Similarly, despite efforts to include more women participants, they were underrepresented among the patient participants. Our participant sampling did not capture the views of people living with DM/HTN who were not attending the surveyed clinics, including refugees, whose perspectives may have been different. We were– to some degree– able to counteract this by asking patients, providers and stakeholders for their insights into the wider communities’ experiences and preferences. Given the study design, we cannot comment on the final health system outcomes included in our NCD model of care conceptual framework, such as the effectiveness or efficiency of the decentralisation project. Finally, the findings may not be generalisable to humanitarian settings or health system contexts beyond Iraq.

## Conclusions

This study suggests that decentralising NCD care to the primary level in a humanitarian setting may be acceptable to patients, staff and stakeholders and can improve access to care. We identified important factors influencing its implementation and sustainability, such as ensuring the continued provision of key health system inputs, especially a continuous supply of good quality medications, and the role of patients’ trust in the service. The lessons learned by the ICRC and the DoH may be useful for other regions in Iraq and elsewhere that are planning to decentralise NCD care to the primary level. Our findings may also encourage actors to explore participatory approaches that put the patient and community at the centre of intervention design and implementation.

## Supplementary Information

Supplementary Material 1.

Supplementary Material 2.

Supplementary Material 3.

Supplementary Material 4.

Supplementary Material 5.

## Data Availability

The datasets used and analysed during the current study are available from the corresponding author on reasonable request.

## Abbreviations

DM: Diabetes Mellitus
DoH: Department of Health
DRC: Danish Red Cross
EML: Essential Medicines List
HbA1c: Glycosylated Haemoglobin
HIV: Human Immunodeficiency Virus
HMU: Hawler Medical University
HTN: Hypertension
ICRC: International Committee of the Red Cross
IDPs: Internally Displaced Persons
KRI: Kurdistan Region of Iraq
LMIC: Low- And Middle-Income Countries
LSHTM: London School of Hygiene & Tropical Medicine
NCDs: Non-Communicable Diseases
NGOs: Non-Governmental Organisations
PHCC: Primary Health Care Centre
WHO: World Health Organization
