# Supplementary material for: Decentralising healthcare for diabetes and hypertension from secondary to primary level in a humanitarian setting in Kurdistan, Iraq: a qualitative study
Source: BMC Health Serv Res. 2025 Apr 15;25:548. doi: 10.1186/s12913-025-12571-6 (PMC11998334; doi:10.1186/s12913-025-12571-6)
Supplement: Supplementary file 1 — Supplementary Material 1. [file 12913_2025_12571_MOESM1_ESM.docx]

**Supplementary File 1: Conceptual Framework Definitions**

| **Conceptual Framework Domains** | **Domain Definitions** |
| --- | --- |
| **Context** | **Humanitarian and country socio-economic, cultural and policy factors**  Host country socioeconomics; context causing humanitarian crisis; sub-populations e.g. host population and displaced persons; relationship between patients and service providers (i.e. trust); Politics/culture/industry around food, exercise, health beliefs & health care.  Social protection; Public policies around food/ exercise/ food industry/ smoking/ alcohol ; Availability of culturally appropriate exercise options; smoking & alcohol education /cessation; Humanitarian policies and funding |
| **Crisis Phase** | **Acute crisis and acute emergency response:** any disaster, whether natural or human-made, can become a humanitarian emergency if international humanitarian assistance is needed to support the affected population**. The emergency response** begins immediately after disaster strikes, often characterised by population movement. Humanitarian organisations begin to respond, focusing on providing critical services such as food, water, sanitation, primary healthcare, and shelter. Priority is preservation of life.  **Protracted crisis:** conflict or displacement lasting several years  **Chronic/cyclical crisis:** crises persisting or recurring over decades or generations |
| **Required health system inputs** | **Leadership/governance:** NCD health care policy frameworks (national & humanitarian systems); oversight/accountability, coalition building, regulation, incentives, system design.  **Financing:** funds for NCD care (including self-care); humanitarian financing  **Facilities and services:** effective, safe, good quality health interventions for whom, when and where needed with minimum waste of resources.  **Medicines/Equipment:** equitable access to essential drugs & delivery devices; good quality, safe and effective diagnostics; supply chain; drug procurement.  **Health workforce:** responsive, fair, culturally appropriate and efficient  **Information:** production, analysis, dissemination and use of reliable and timely information on NCDs; M&E; patient education; workforce training; guidelines |
| **Patient-centred factors** | **Knowledge:** knowledge of the health condition, risk factors, health services  **Education:** patient education level  **Preferences and Trust:** choice of providers; Perception of services: e.g. quality, trust and value placed in service  **Distance:** distance to services  **Income:** household income and assets |
| **Intermediate health system goals** | **Access and coverage:** availability, accessibility, accommodation, affordability, acceptability  **Quality:** quantity of care; clinical quality; acceptability; convenience; interpersonal relations; patients’ trust in the service.  **Safety:** processes to avoid, prevent & ameliorate adverse outcomes / iatrogenic injuries; adequate, consistent supply of appropriate quality medications; adequate referral pathways.  **Responsiveness:** autonomy, choice, communication, confidentiality, and dignity, quality of basic amenities, prompt attention, and access to family and community support.  **Integration:** integrated service delivery is the organisation and management of health services so that people get the care they need, when they need it, in ways that are user-friendly, achieve the desired results and provide value for money  **Continuity:** the process by which the patient and his/her physician-led care team are cooperatively involved in ongoing health care management toward the shared goal of high-quality, cost-effective medical care. Three types of continuity in every discipline: informational, management, and relational.  **Standardisation:** development, implementation and evaluation of shared, unified tools such as Standard Operating Protocols (SOPs), guidelines and protocols, to implement best practice and limit local variation in NCD care delivery  **Sustainability:** In relevant contexts, such as situations of chronic vulnerability, effective humanitarian action requires collaboration beyond the humanitarian community, away from cycles of short-term delivery and toward a sustainable framework of human rights and social protection; reinforcing rather than replacing existing capacities and coping strategies; collaborating to reduce and end humanitarian need |
| **Final health system goals** | **Improved health; social and financial risk protection; improved efficiency:** A health system and parallel humanitarian health system responsive to the DM/HTN needs of refugees/IDPs and the host community; evidence-based services leading to improved health; reduced rates or severity of complications; better quality of life and improved functioning. Access to care balanced with provider quality and safety. |
