# Supplementary material for: Decentralising healthcare for diabetes and hypertension from secondary to primary level in a humanitarian setting in Kurdistan, Iraq: a qualitative study
Source: BMC Health Serv Res. 2025 Apr 15;25:548. doi: 10.1186/s12913-025-12571-6 (PMC11998334; doi:10.1186/s12913-025-12571-6)
Supplement: Supplementary file 2 — Supplementary Material 2. [file 12913_2025_12571_MOESM2_ESM.docx]

**Supplementary File 2:** **Topic Guides**

**Indicative Topic guide – Semi-structured interviews with DM/HTN patients or carers**

| **Key area** | **Themes** | **Question** |
| --- | --- | --- |
| **Introduction** | Study aim and agencies involved  Why invited to participate  Consent & any questions? |  |
| **Participant Background** | Getting to know each other & building rapport | **Could you tell us a bit about yourself?**   - *Prompt: e.g. Ask about age, where they currently reside, their family circumstances and if appropriate, current work/study. Where were they living before/ previous work/study/ education level?  How long living in Duhok, with family or not? Have you moved within Duhok?  (cultural background/languages spoken)* |
| **Patient Pathway - Diagnosis** | Diagnosis  Initial care seeking and supports  Prior knowledge and knowledge in community | **What medical condition or conditions are you (or your family member) attending care for in Duhok?**   - *Prompt – If more than one condition or complication, ask about each separately*   **What made you (or your family member) look for help for this issue/problem/illness?**   - *Prompts – feeling unwell? What symptoms? When did symptoms start e.g.in Duhok or in home place/ country?* - *Prompted by family member or friend’s suggestion, by knowing someone who had similar symptoms, community worker, messaging via radio, television, internet?*   **Where did you go for help?**   - *How long ago did you seek help, from where? (4Ws, what links between different levels/sites of care?)* - *Was there a delay in seeking help and, if yes, what was the reason? E.g. cost, distance, knowledge, social constraints* - How did you feel when you were first diagnosed? (*anxiety, worry, fear, relief, acceptance, anger etc. )*   **Did you know about [diabetes/HTN] before you (or your family member) were/was diagnosed? What did you know about it?**   - *Prompt –* source of knowledge (incl. internet, radio, family members, community etc; - *Knowledge about cause, prevention, treatment, prevention and treatment of complications, implications for future.*   **What information did you (or your family member) initially receive after you/they were first diagnosed?**   - *Prompts – what kind of information, by whom, how (verbal, written material, web resources, support or community group)* - *Did you find it useful/appropriate/did it make sense to you?* - *Did you look for extra information from other sources (e.g. internet, family, pharmacist, TV etc)?* - *How do you know if the information is correct, reliable or can be trusted?*   **Could you tell me about what people in your (x) community know about (diabetes/HTN)? Are people familiar with it?**   - *Prompt - among family, neighbours, friends, children; type of knowledge and sources of knowledge as above)*   **Where do people in your community with NCDs get medications for their conditions?**   - *Prompt – primary care centre, private doctor, hospital, pharmacist, friends/family* - *How do they find out about these resources, get to them, pay for them? What are the barriers –knowledge, transport, cost, social constraints*   **Can you tell me about how other people in your community get medical advice or consultation for NCD conditions e.g. blood pressure or blood glucose checks and medication adjustment?**   - *Prompt – primary care centre, private doctor, hospital, pharmacist, friends/family;* - *How do they find out about these resources, get to them, pay for them? What are the barriers –knowledge, transport, cost, social constraints* |
| **Patient Pathway – Current care** | Current services and support  Community based care  Access and Covid | **What health services or supports do you (or your family member) currently use to help you with your condition?**   - *Prompt – What health facility do you attend and what is offered – 4 Ws; compared to any previous care in Duhok or where you lived before?* - ***If the person is an IDP, probe for how they managed their condition during conflict, flight and when they arrived in Duhok. How did they know where to seek care?*** [if decentralisation from Gulan to PHCC and back is mentioned skip ahead to next section]   **If these specific items are not already mentioned, probe for them:**   - ***What*** ***medications do you take*** *(i.e. Indication not name); How do you get/pay for them? Health facility/other source(s), How do you manage your meds regime (of insulin if relevant) adherence and support, gaps in supply* - ***What equipment do you use;*** *(e.g. Glucometer, lancets, syringes, needles, BP cuff, exercise equipment)/ appropriate? Where do you get/ pay for these? Any difficulties with accessing or using these?* - ***What lab tests or medical checks do you do****? (e.g. Blood tests, foot check, eye check, ECG) Where, how often, cost, any access difficulties?* - ***What kind of healthy living education or programme do you get*** ***(note*** *will be focused on in a later question)* - ***What kind of referral services do you use/ have access to*** *(secondary/tertiary care, MHPSS, physio or rehab, protection services, link to other refugee services?)* ***What about eye checks, kidney checks, foot checks in Diabetes.***   **How easy or difficult is it to access these services?**   - *If not covered, discuss what makes it easy/ difficult? (Are there any issues around* *cost, eligibility, availability, physical distance/ transport, social commitments or barriers)*   **How have health services/supports changed because of Covid-19?**   - *Change in services offered, frequency or type of contact, cost, physical access, restrictions, transport.* - *Any particular fears around Covid, Have you changed your own behaviour in any way?*   **Are you (your family member) accessing any help outside of the health system at the moment?**   - *Pharmacist; community-based groups, charities, volunteers, formal/informal (e.g. Syrian doctors), non DM/HTN related?* - *How easy/difficult to access these – find, get to, pay for* |
|  | Daily routine  Healthy living  Barriers and facilitators to self-care  Wellbeing  Complications | **Could you tell me about your (or your family member’s) daily routine around managing your/their [diabetes/HTN]**   - *Daily routine, nutrition, physical exercise, impact on daily living, family life, work life; functionality ADL/work/school* - *Symptoms (any pain)? Effect on quality of life?* - *Challenges (knowledge, adherence meds/ lifestyle, stigma, worries, beliefs about the illness; financial impact* - *What helps you to look after your [Diabetes/HTN][if not covered above]? Current supports / additional supports needed?  (family, friends, community-based, financial, psychological supports, access to healthy food /exercise options, tailored advice, rehab/ physio/palliative care)*   *Do you get* ***any support from friends and family to take care of your [diabetes/HTN]?*** *[if not covered above]*   - *Medication pick up/reminders/management/ food preparation/ exercise/ accompanying to appointments*   **What is the main concern for you (or your family member) in managing your/ their illness?**   - *How to find /pay for medications; finding enough healthy food; finances; employment; finding/paying for good quality, affordable medical consultation/ referral for specialist opinion/intervention*   **How does having diabetes/HTN affect you (or your family member) emotionally? How do you deal with your concerns/worries?**   - *Prompt - Does the condition affect your mood and sense of well-being?* - *Cause you to feel upset or cause stress? Sources of support if you feel upset or stress/ worried/ anxious? Do you talk to anyone? (Family, friends, neighbours, community, CHW, HCP, religion, books, internet).* - *Specific services or supports available for people who are distressed? (Counselling, psychology, psychiatry; what supports/services, where, by whom, how to access?).  Supports offered by health care worker/CHW?* |
| **Quality/ Patient-centeredness of current care** | Access and coverage  Experience, respect and dignity  Communication  User focus  Quality of infrastructure | **If attending more than one service** (e.g. health centre, hospital, pharmacist, support group, community volunteer programme), **ask in relation to each and to services accessed elsewhere in Duhok. Focus on differences between PHCC and**  **What do you think about the services you are currently receiving for your HTN/DM?**   - *Prompt – quality, sufficient, acceptable – type, amount (frequency, time offered), location, quality, trust, appropriate to you/family member and refugee status (if appropriate) – meeting expectations?* - Perceived competence, numbers, and training of providers, medicine, and equipment. - What about specialists or investigation e.g. heart specialist, diabetes specialist, kidney specialist, eye screening, and angiogram. - Physical access (distance, transport cost /availability, physical/cultural/security impediments); Language; Accommodation (appointment system, waiting times, flexible opening hours, walk in if urgent) - Cost (any payments including outside of facility; how paid; affordability vs. household income, choices made)   **What has your (or your family member’s) experience of DM/HTN services/care/support been like?**   - *Prompt - if attending more than one service/location, ask this about each]* - *How did you feel about the care/treatment you were given? (Satisfied, trust in service and providers; if unsatisfied: probe for changes desired]*   **How did you feel you (or your family member) were/was treated by staff?**   - *Prompt – respect, dignity, privacy, non-discrimination, autonomy, confidentiality* - *Clear explanation, good communication e.g. time to ask questions, questions satisfactorily answered, speaks slowly, verifies what patient understood, accessible language, use of mother tongue]* - *If you had questions about your treatment or an emergency do you have a point of contact?*   **Can you tell me about whether you have been involved in decisions about your (your family member’s) health care or treatment?**   - *Prompt –asked for their input or opinion, given options to choose from, asked re concerns, family included in discussion* - *Offered choice of provider, info re other types of services for condition, freedom to choose provider/service/treatment*   **What do you think of the physical space or facilities where you (your family member) are/is receiving care?**   - *Prompt – privacy, confidentiality, comfort, cleanliness quality of clinic room/ waiting room (space, seating, fresh air)*   **If relevant, how does the current service at the PHCC compare to when you were going to Gulan centre?**   - *Prompt – convenience, physical access, distance, perceived quality, available services, cost, trust, comfort*   *How have things changed during Covid restrictions (March to May) and since then?* |
| **Integration and continuity of current care** | Integration  Continuity of care  Covid | **Do you have regular contact with the service(s) you (or your family member) attends?**   - *Prompt –how often, appointment based? Access outside appointment times? What do you do in an emergency or if you are feeling unwell? Are there appointment reminder systems?* - *Has this changed during Covid? How were things before/during the restrictions from March to May? How about now?*   **If you need to go to different health centres/hospital/doctors/pharmacist (using terms interviewee has used previously), how does that link up in terms of information?**   - *Prompts – Information - how is information passed on about your diagnoses, what medications you take or about your blood pressure or diabetes control?  -Is there a clinic or patient held paper file; any electronic records; is information shared with other services e.g. community/referral/ pharmacist; if moving within Duhok? Do you have to share the information yourself?* - *Continuity of clinical management - do you feel that you receive consistent advice and approach to clinical management of your diabetes/HTN? Or do different providers tell you different things?* - *Continuity of relationship  - same health worker/ team at the same facility/ community level*   **How do the current services support you (your family member) to manage your multiple conditions (if relevant)?**   - *Probe: Adapt advice/ services to multiple symptoms/ functionality/ medications?  Manage all conditions / refer? Different providers for different conditions? How do you personally manage (symptoms, treatments, providers)?* |
| **Thanks and**  **close** | Anything else to add  Questions/Thanks | **Is there anything else you could suggest that would improve how you look after (take care of) your diabetes/ HTN?**   - Can you think of any new or different approach to managing your DM/ HTN that might be helpful for you (or others)?   **Is there anything else you would like to add? Is there anything you think I should have asked you about that we haven’t talked about?**  **Do you have any questions for me?**  **Are you all still happy to have your data included for this evaluation?**  **Thank you for your time.** |

**Indicative Topic guide – Semi-structured interviews with DM/HTN providers**

| **Key area** | **Themes** | **Question** |
| --- | --- | --- |
| **Introduction** | Study aim and agencies involved  Why invited to participate  Consent & any questions? |  |
| **Participant Background** | Getting to know each other & building rapport  Understanding provider’s role and experience | **Could you tell us about your current role in the health centre? Could you describe your typical working day?**   - *Prompt : tasks, workload and workflow,* - *Prompt: length of time working here, in this role? Working anywhere else currently or recently?*   **What is your role in relation to diabetes and hypertension (DM/HTN) care?**   - *Prompt: tasks in relation to NCD care, interaction with patients* - *I understand there have been some changes in how NCD care has been provided/organised in the last few years (decentralisation/patient transfer from Gulan hospital to the PHCCs) Have you been aware of this? Has this affected your role/work?* - *Prompt – change in your responsibilities, tasks or type of work? Workload change (more or less busy)?* |
| **Patient Pathway – Diagnosis and patient pathway** | Diagnosis  Initial care seeking and supports  Care pathway  Community based care  Knowledge and care seeking in community | **Who is your [organisation /service] providing services for?**   - *Prompt – IDPs, Syrian refugees, other refugees, vulnerable host population, host community in general, specific groups e.g. women/elderly*   **In general, what level of knowledge do you think the community/ general population has around chronic diseases such as DM/ HTN? (differentiate if needed between IDP/local population/other groups in community, as identified above)**   - *Prompt – What do they know about the disease, its prevention and its treatment? Do they know what symptoms to look out for? Do they know where to go for medical care if they suspect they have diabetes/ hypertension?* - *Where do they get their information?  Family, community, TV, radio, internet, smart phone, community workers, health care providers, pharmacists, informal sector.* - *Do you know about any education campaigns, awareness raising or interventions around healthy living and chronic disease prevention for the population/community in general?*   **How are people identified or diagnosed as having diabetes or hypertension? How do they find out they have these condition?**   - *Prompt - referral from other services, opportunistic screening at PHCC [e.g. BP check of everyone >40yrs] or community-based screening, community awareness raising, self-referral* - *If identified at community level, how do they reach the PHC for confirmation of diagnosis and or initiation of management*   **What happens next for the patient following their diagnosis? How are they eligible to receive health services for diabetes/hypertension at the PHCC?**   - *Prompt – enrolled in services? How are they followed up? patient pathway: two-way referral between system levels/ sites; links to community services; individual/group counselling/interventions; medication adherence support, supported self-care, primary care functions; secondary or tertiary care functions; specific services children/ women; family support, rehab/ physio, palliative or home-based care*   **Are there any other supports or information available for people with diabetes/ hypertension?**   - *Prompts – kind of information, by whom, how (verbal, written material, web resources, support or community group)* - *Useful/appropriate/comprehensible? Other sources of information (e.g. internet, family, pharmacist, TV etc)?*   ***At community level, are there any kinds of programmes or interventions for people who have already been diagnosed with HTN/DM?***   - *Prompts****:*** *peer support groups, MHPSS, livelihood interventions, home based care, community nursing, rehab/ palliative care* |
| **Patient Pathway – Current care** | Current facility-based care and support | **What services are provided by your facility or organisation? [If not already covered above]**  **Have these changed since [decentralisation/transfer from Gulan]? In what ways?**   - *Providing medications. Do patients get specific adherence support/counselling;* - *Investigations/Labs? Complications screening and management (lab tests - which ones? – retinopathy screening, diabetic foot check, ECG)* - *Healthy living education or intervention* ***(note*** *will be focused on in a later question)* - *Referral services (secondary/tertiary care, MHPSS, physio or rehab, protection services, link to other refugee services?*   **Can you tell me more about the numbers and types of staff involved in DM/HTN care at the PHCC?**   - *Prompt - as relevant, numbers of: community volunteers/health workers/ peer supporters; specialists, generalists, nurses, care assistants, educators, counsellors, pharmacists, nutritionists;* ***number of patients seen a day; time slot for each patient; sufficient time to address/respond to patient need*** - *Has this changed since [decentralisation]? In what way?*   **Do you feel you have the training you need to deliver NCD care? Has this changed since [decentralisation]? Could you tell me about any training you received in DM/HTN care?**   - *Prompt -* Undergraduate, specialist training, specific course, refresher training? - Specific to [decentralisation] process? Who was it provided by? Did you find it useful? Is there anything else it would be helpful to have training about?   **Do you feel you have the support or supervision you need to deliver NCD care? Has this changed since [decentralisation]**   - *Prompt – are the any challenges, anything you would change or any areas you feel it would be helpful to have more support or training on?*   **Could you tell me about any specific written protocols, guidelines, standards, or regulations being used for DM/HTN management services and care [by your organisation/at this facility]?**   - *Prompt - name of protocol or guideline, where it is from?* - *Is it useful? Is it used by staff? Why/why not?*   **Could you describe other tools you use?**   - *Prompt - Education materials – written, leaflets - Are these useful? Do patients understand them?* - *Equipment – what kinds of equipment? Does it work reliably? Does everyone know how to use it?* - *mobile technology; supplies, medicines, and information systems; culture of quality control and audit, use of data collected, supervision, and feedback; any lack of tools*   **Are there any problems with medicine supply, stock outs, or medicines quality? [patients have mentioned this as an issue]** |
|  | Access  Complications  Barriers and potential facilitators to care for those with complications | **Do you think there are any challenges for patients to access health services for their diabetes/hypertension?**  **Thinking about different groups in the local community - is it easier/harder for some groups than others? (older/young, women/men, IDP/local community/other displaced groups)**   - *Prompt - sufficient, acceptable – type, amount, location, quality, trust - appropriate to needs, preferences and knowledge.* - Adequate referral services - Physical access (distance, transport cost /availability, physical/cultural/security impediments); Language; Accommodation (appointment system, waiting times, flexible opening hours, walk in if urgent) - Cost and affordability – can patients afford to pay for services and medications? is the 500(x) charge affordable for everyone?   **Are you involved in providing patient education about healthy living, diet and exercise? What do you tell them? How do they respond?**  **What kinds of things do patients do to help manage their conditions themselves at home?**   - Prompts: adapting their diets? Monitoring blood pressure or blood sugar at home? Exercise? - Do you have any suggestions for how patients could be supported to do that?   **What about patients with complications? Are there different challenges for them? How does the system work for them?** |
| **Quality/ Patient-centeredness of current care** | Quality  Experience, respect and dignity  Communication  User focus  Quality of infrastructure | **Do you think the current services meet patients’ expectations?**   - Prompts - Perceived competence, numbers, training of providers, and quality of infrastructure, medicine, and equipment. - DM/HTN services adapted to socio-cultural needs of refugees/host community, delivered in native language, meeting expectations of care and preferences; stigma surrounding help seeking. - **What do you think are the important aspects of the service from the patient perspective? What do they value?** - *Prompts -* *evidence-based, effective care: systematic assessment, correct diagnosis, appropriate treatment, counselling, and referral; appropriate lifestyle advice and education materials – adapted to the context; capable systems: safety, prevention and detection, continuity and integration, timely action*   **What do think is the patients’ experience of care and their trust in the service and health care providers?**   - *Prompts -* *Respect: dignity, privacy, non-discrimination, autonomy, confidentiality, and clear communication; user focus: choice of provider, short wait times, patient voice and values, affordability, and ease of use; satisfaction, recommendation, trust care, uptake and retention*   **Do you think patients would like to see anything done differently?**  **What do you think of the package of care for NCDs (DM/HTN) provided at the facility? How does it compare with what is available elsewhere (including in the private sector?)**  **Can you think of any strategies, which might improve the way hypertension/diabetes care is provided?** *Prompt - train lay health care providers; involve service-users in provision of care; better patient education; reorganisation of care/efficiency; integration of DM/HTN in PHC/other platforms of care; information sharing between sites/levels; funding and access to investigations and interventions*  **If you could make a recommendation or give feedback to people doing the same [decentralisation] process elsewhere in Iraq, what would it be?** |
| **Integration and continuity of current care** | Integration  Continuity of care | **Can you describe how the PHCC communicates with other levels of care?**   - *Prompts - Communication and continuity between community (CHCW/self-help or peer-support groups; pharmacist etc.) and PHC (in each direction); community outreach (mobile clinic, community-based nurse etc.);* - *Referrals to secondary or tertiary care/other services such as MHPSS, health education and outcomes;* - *How successful is the referral pathway, challenges in terms of continuity, communication, confidentiality of data; potential solutions*   **How are patients followed up - ensuring regular attendance at the PHCC?**   - *Prompts - appointment systems, reminders, defaulter tracing, community outreach, registration book, patient held/clinic-based file, what data collected/ shared with other providers, what referral system is in place and how well does it function* - *How often seen, appointment based? Access outside appointment times? Recall or reminder systems?* |
| **Thanks and**  **close** | Anything else to add  Questions/Thanks | **Is there anything else you could suggest that would improve management of DM/HTN for your patient population?**  **Can you think of a new or innovative approach to managing you DM/ HTN?**  **Is there anything else you would like to add? Do you have any additional questions for me?**  **Are you all still happy to have your data included for this evaluation?**  **Thank you for your time.** |

**Indicative Topic guide – Semi-structured interviews with DM/HTN Key Stakeholders**

| **Key area** | **Themes** | **Question** |
| --- | --- | --- |
| **Introduction** | Study aim and agencies involved  Why invited to participate  Consent & any questions? |  |
| **Participant Background** | Getting to know each other & building rapport  Diagnosis | **Could you please describe your current role and your involvement in providing diabetes and hypertension (DM/HTN) services in Duhok?**  **In general, how are people with hypertension and diabetes diagnosed with these condition(s) in Duhok? How do they access care in Duhok?  What about people who are IDPs/refugees? Any differences in access or use of services for men/women?**   - *Prompt -* ***What services are available:*** *prevention, detection, education, medical management, referral, rehabilitation, and palliation –* ***Private vs public***   **How has Covid affected the provision of NCD services in Duhok? How if at all has patients’ access to NCD care been affected by Covid?**   - *Prompt – effects for diagnosis, consultations, medication, any particular patient groups more/less affected?* |
| **Patient Pathway** | Diagnosis  Care pathways | **IF INVOLVED IN DECENTRALISATION OF NCD CARE:**  **We are particularly interested in the policy of decentralisation of NCD care, that is transferring care of DM/HTN patients from hospital to PHCC level, that ICRC and the Duhok DOH have been working on in the last few years.**   - **[***If relevant***] Are you aware of this policy/programme?**   **What has your/your organisation’s role been in decentralisation of NCD care to PHCC level?**   - *[If relevant]* **Which services were offered by your organisation, where, by whom, for whom before decentralisation?** - *Prompts - what conditions? What services: education; medication, consultation, lab tests – which ones, complications screening/management: ECG, X-ray, Echo, diabetic foot care, ophthalmoscopy, retinal screening and laser therapy, dialysis; MHPSS, rehab/physio, palliative care, home care for house-bound patients.* - *Is there any differences in service provision for IDP and for the host community?*   **Have your services changed in any way because of the decentralisation of NCD care? Please tell me more about that. Were there any challenges with decentralisation?**  **Did the policy fit in with the existing services and guidelines? Did any adaptations need to be made for decentralisation to happen?  Please tell me more about that.** |
|  | Policy and guidelines  Training and support | **What do you think was the goal of the decentralisation policy? Did it achieve that goal? Could you tell me more about that?**  **What resources were devoted to the policy/programme of decentralisation?**  **Were there any resource issues in terms of staffing, work pattern, workload that impacted on your ability to implement the decentralisation policy/programme?**    **Could you tell me about any training or guidance that relevant staff receive in NCD care in general? Was any additional training or guidance given around the decentralisation policy/programme?**   - *Prompts -* *undergraduate, specialist training, specific course, refresher training, or specific to needs of refugees)*   **What methods of follow-up support were put in place after the policy was introduced? How effective were these? Are there any changes you would make to make these more effective?**   - *Prompts -* *regular supervision, case discussions, responsive point of reference for queries* |
| **Integration and continuity of current care** | **C**ontinuity of care  Referral pathways | **Could you tell me about the process of transferring the care of selected patients to PHCC level?**  **How were people with HTN/DM identified as being suitable to have care delivered at PHCC level rather than at hospital level?  Which patients were retained at hospital level and why?  Did this selection of patients work/not work?**  **Could you tell me about how patient information was transferred between sites?**  **How did continuity of care work for those who were transferred?**   - *Transfer of patients’ files/data; challenges in terms of continuity and communication; any challenges noted around confidentiality of data; potential solutions to challenges identified?*   **Is there a pathway and set of criteria for patients to be referred back from PHCC level to Gulan hospital? Could you tell me more about that? How well does the referral pathway work?**   - *Data sharing, continuity of information, bidirectional flow of information?*   **What have been the successes and challenges in terms of communication and continuity of care between PHCC and hospital level?**   - **[***Ask now or later when talking about community services***] What about integration with other providers offering services relevant to people with NCDs? For example, services at community levels or tertiary care level?** - *Communication, referral pathways, funding, accessibility, affordability; potential solutions to identified challenges* |
|  | Access  Barriers and potential facilitators to care | **Are there any issues for patients who have had their care transferred to the PHCC in terms of access to PHCC services?**  **Are there any issues for these patients to access hospital or specialist care? Has decentralisation affected this?**   - *Prompt – sufficient services* Physical access Accommodation Cost and affordability   **Have there been any unexpected consequences for patients in terms of access or convenience?**  **Can you think of any strategies to provide greater access to care or greater continuity of care?** |
| **Quality/ Patient-centeredness of current care** | Quality  Experience of care  Acceptability | **How acceptable do you think the policy of transferring HTN/DM care to PHCC level is to patients?**   - *Prompts -* *meeting their expectations of care and preferences?*   **What do think is patients’ experience of care and their trust in [your organisation’s services]? Has there been any change in this since decentralisation happened?  What about trust in other available NCD services?**   - *Prompts -* *Respect: dignity, privacy, non-discrimination, autonomy, confidentiality, and clear communication; user focus: choice of provider, short wait times, patient voice and values, affordability, and ease of use; satisfaction, recommendation, trust care, uptake and retention*   **How would you describe the quality of care for DM/HTN that patients receive at PHCC level compared with at the hospital level?**   - *Prompts -* *evidence-based, effective care: systematic assessment, correct diagnosis, appropriate treatment, counselling, and referral; appropriate lifestyle advice and education materials – adapted to the context; capable systems: safety, prevention and detection, continuity and integration, timely action*   **Do you have any particular concerns about the quality or safety of services at PHCC level? What about at hospital level?**  **What have been the successes in terms of how the decentralisation policy has been implemented? What have been the challenges? What could be done to improve these issues?**  **The Ministry of Health in Baghdad is interested in implementing a similar decentralisation policy elsewhere in Iraq. What advice would you have for the Minister about implementing this policy?** |
|  | Knowledge and care  in community  Community based care | **IF EITHER INVOLVED OR NOT INVOLVED IN PROVIDING CARE AT COMMUNITY LEVEL:**  **We are also interested in care or services that are provided for people with DM/HTN at community level.**    **From your experience, could you describe the current knowledge of the population in Duhok around chronic diseases such as DM/ HTN?**   - *Prompts - do they know where to seek care and what is available?* - *Same for everyone or different for particular groups in the population?* - *Disease knowledge – disease course, risk factors, complications; sources of knowledge – family, community, TV, radio, internet, smart phone, community workers, health care providers, pharmacists, informal sector.* - *Population level education, awareness raising or interventions around healthy living and chronic disease prevention*   **Does your organization provide education, awareness raising or prevention services for DM/HTN? Could you describe them?**   - *Prompts - primary prevention e.g. outreach efforts, community-based activities, support for behaviour change etc. Also probe for screening and secondary prevention, like nutritional counselling, foot care for diabetics, etc.  Ask for copies of educational material.*   **Can you tell me about patient access to these services? How do patients find them?**   - *Prompts -* *meeting their expectations of care and preferences* - **Could you tell me about the quality of these services?**   **With regards to your organisation’s model of DM/HTN care, what have been the successes, challenges and lessons learned?**  **How well, in general, how well do you think patients with HTNDM are able to manage their own conditions at home?**   - Prompt - key constraints or facilitators; knowledge, beliefs; access and/or affordability of support, services, tools  equipment, medications; motivation; family or community supports; trauma and mental health issues, low priority, stigma - Do you think any particular groups are able to manage more or less well?   **Are there any support available to patients at community level at or to manage their care at home? Could you elaborate on that?**  **Are there any available services for physiotherapy, rehabilitation, nutrition, psychosocial support, palliative care for HTN/DM patients of your services?** |
| **Thanks and**  **close** | Anything else to add  Questions/Thanks | **Is there anything else you would like to add? Do you have any additional questions for me?**  **Are you all still happy to have your data included for this study?**  **Thank you for your time.** |
