# Supplementary material for: Decentralising healthcare for diabetes and hypertension from secondary to primary level in a humanitarian setting in Kurdistan, Iraq: a qualitative study
Source: BMC Health Serv Res. 2025 Apr 15;25:548. doi: 10.1186/s12913-025-12571-6 (PMC11998334; doi:10.1186/s12913-025-12571-6)
Supplement: Supplementary file 3 — Supplementary Material 3. [file 12913_2025_12571_MOESM3_ESM.docx]

P4C Iraq **Provider** Coding Framework: Initial themes

*These are mainly descriptive themes to group the data from the transcripts. They reflect the main topics that were discussed in the interviews (which is a product of what was suggested in the topic guides, what was asked to patients, and what patients responded to/wanted to talk about). Some will break down further, e.g. into challenges, positive/negative experiences etc. Our interpretation of the data in those groupings will follow as we code. We will then come back to the conceptual framework which guided the topic guide and look at how the domains identified from the data relate to those domains.*

1. Role of person at the health centre or typical workday
2. Local knowledge and awareness of condition
   1. Content
   2. Sources (family and friends, posters, health services; TV; not radio or internet)
3. Community-level services (or lack thereof)
4. Services at local health centre
   1. Description of services (available/not available)
      1. Diagnosis
      2. Equipment (including lab testing)
   2. Health education
      1. Content (diet or exercise, taking medicines on time)
      2. Approach, e.g. verbal or supportive materials; lack of specific education sessions
      3. Perceived patient adherence to advice
   3. Changes to services over time
   4. Description of patient flow
   5. Processes for follow-up and referrals and referral criteria (written referral letters; referring those whose BP, BSL is uncontrolled)
   6. Staff
      1. Number and roles of staff
      2. Training and education
         1. Related to decentralisation, content, who delivered it (ICRC)
         2. Related to NCDs
      3. Confidence in delivering care
   7. Information systems
      1. Patient clinical data, e.g. chronic disease booklet
   8. Recall/appointment systems (e.g. phoning those who miss appts)
   9. Quality and responsiveness
      1. Time spent with each patient and patient numbers
      2. Waiting, appointment system, and opening times
      3. Clinical guidelines
      4. Complaint mechanism, languages
   10. Access to health centre (incl. whom services is provided for; appointment times or not)
5. Medicines
   1. Medicines at local health centre (available, non-available; quality)
   2. Differences in medicines between PHCC & Gulan
   3. Perceived patient’s attitude to medicines (e.g. unsealed medications, valuing medicines above all else)
   4. Interrupted medicines supply and patient solutions
   5. Prescription of medicines in Gulan that are not available in PHCC
6. Services at Gulan hospital
   1. Description of services
   2. Information (patient card)
7. Comparisons between PHCC/Gulan Hospital
   1. Different staffing
   2. Costs
   3. Services, e.g. creating booklet
   4. Transport/distance/accessibility
   5. Perceived quality from staff perspective; from patient perspective (medicines, health professionals, etc.)
8. Comparisons between PHCC and other services, e.g. private care
   1. Affordability
   2. Perceived quality and availability (e.g. of medicines, equipment)
9. Service changes during/since decentralisation (during and after ICRC support ceased)
   1. No changes since decentralisation
   2. Training (NCDs, paperwork, medicines supply) & Ongoing support and supervision
   3. Number of patients
   4. Provider workload (PHCCs vs Gulan)
   5. Services and medicines
      1. Addition of HbA1c
      2. Some services continue to be in Gulan (e.g. for CVD), some in PHCC
   6. Medicines supply chain
      1. Integration with existing supply chain, medicines lists, pill burden, combo medications, and physician practice/guidelines
      2. Challenges, Stock outs and reasons for this
      3. Cost, implications for staffing at PHCC
10. Perceived patient expectations/ experience
    1. Perception of decentralisation
    2. Perception of quality at Gulan vs. PHCC
11. Provider’s suggestions for improvements/changes to services
    1. Staff incentives; increased number of staff
    2. More lab equipment and better-quality medicines
    3. Training on NCDs (to inc. Dispensing staff)
    4. Better coordination of medicines list and supply chain (suggest a coordination committee, including physician prescribers to unify the order)
12. Cross-cutting themes
    1. Perceived responsibility for care
13. Changes due to Covid
