# Supplementary material for: Decentralising healthcare for diabetes and hypertension from secondary to primary level in a humanitarian setting in Kurdistan, Iraq: a qualitative study
Source: BMC Health Serv Res. 2025 Apr 15;25:548. doi: 10.1186/s12913-025-12571-6 (PMC11998334; doi:10.1186/s12913-025-12571-6)
Supplement: Supplementary file 4 — Supplementary Material 4. [file 12913_2025_12571_MOESM4_ESM.docx]

Iraq **Key Stakeholders** Coding Framework: Initial themes

1. **Role of key stakeholder**
   1. General role
   2. Within decentralization project/policy
2. **General comments on decentralization**
   1. Role of the different actors and their relationship
   2. Scope of the project
      1. Limited to one district
      2. Focus on DM/HTN because of disease burden and insulin through NN
   3. Value of project
      1. Being a good idea
      2. Too short duration of project (two years)
      3. Achievement of objectives
   4. Reason for implementing project
      1. ICRC’s idea and supported by DoH
      2. DoH idea initially
   5. Aim of project
      1. To decrease the load in the hospital/ tertiary center
      2. To improve services at primary health care
      3. To increase access to care or reach
   6. Expansion of project
      1. Interest by DoH to expand to other areas
3. **Description of the process of implementing the decentralization project/policy**
   1. Preparation before or when implementing policy
      1. Training of data collectors at PHCCs
      2. Short training at PHCCs for doctors and for other staff
         1. Feeling of confidence by staff
         2. Challenges and Adaptation of training - high medical staff turnover
      3. Quality appraisal visits to PHCCs by ICRC
      4. ICRC databases for medicines supply chain planning and health records used
   2. Selection of patients for decentralization
      1. Based on chronic disease ID
      2. Selection of facilities PHCC facilities that were closest to Gulan
      3. Referral criteria existing
         1. Application of referral criteria uncertain
   3. NCD service provision
      1. Before project implementation
         1. NCD services and medicines only provided at Gulan hospital
      2. After project implementation
         1. Differences in services between Gulan and PHCCs
            1. Less medicines availability in PHCCs and earlier shortages than in Gulan d/t decision to prioritize supply to Gulan
            2. More specialists in Gulan
         2. Role of doctor at PHCC
            1. Prescribing minor changes to medicines
            2. Transfer paper to Gulan in case patient remains uncontrolled for further medicines changes
            3. Provide monthly medicines
         3. Role of Gulan hospital
            1. Changes to medicines for uncontrolled patients
            2. Transfer back to PHC for patient to collect monthly medicines
         4. Medicines provided
            1. Medication list - (decision making, ICRC providing basic set of medicines, pros and cons of this)
            2. DoH covering additional medicines
         5. Additional services
            1. Some lab tests added
         6. Missing services
            1. Teaching/ coaching about food and exercise
            2. Health information materials on NCDs
            3. Investigations sometimes not available at health centre
            4. Lack of community-based services by any actor
            5. Specialized services (rehabilitation, palliative care, nutrition)
         7. Quality of care at PHCCs
            1. Overcrowded/ too high patient-doctor ratio
            2. Low availability of medicines
            3. Monitoring of quality of care (processes in place)
         8. People seeking care
            1. No differences between IDP, refugee, or host population
            2. Influence of medications availability is major influence on attendance at PHCCs

shortages much more important than presence of doctor in terms of people continuing to attend PHCCs

When another NGO temporarily provides alternative e.g. branded drugs, then patients appear not to attend PHCCs (attendance numbers drop)

1. **Outcome/ impact of decentralization project/policy**
   1. Reported outcomes (positive/negative)
      1. Distance to health facilities reduced for patients
      2. Travel costs reduced because of proximity
      3. Especially valuable for poorer people
      4. Increasing number of patients observed (? attending clinics?)
      5. Perceived patient satisfaction
         1. Increase because services closer to patients’ homes
         2. Increase because ‘original’ medicines from ‘good company’ available for free at PHCC
         3. Decrease, after medicines availability get worse due to hand-over
         4. Decrease, patients do not like the large jars and plastic baggies
      6. Improved clinical outcomes (random informal patient sample)
   2. Challenges
      1. During project implementation
         1. Doctors prescribing medicines not available in ICRC-support health centers (preference for certain drugs, e.g. FDCs, their use of international guidelines)
         2. Medical staff and training
            1. Drs on rural rotation for one year, then leave
            2. Minimal training in primary care/NCDs as med curriculum focused on tertiary care
            3. After training given by DOH (supported by ICRC) at start of decentralisation, turnover of medical staff, lead to loss of knowledge, training
         3. Lack of referral pathway, information sharing pathway between Gulan and PHCCs (reported resistance of DOH and ICRC to invest in that at the time, new system in place now)
      2. After ICRC hand-over
         1. Shortage of medicines
            1. Linked to reduction in # of patients, if medicines unavailable
            2. Medicines are critical as without them nothing can be done
            3. DoH meant to take-over but not having the budget to do so
            4. Issues re Bagdad vs Kurdistan
         2. Fewer patients
         3. No daily patient data (trained data collectors at PHCC not entering data daily; but only monthly through paper-based files)
         4. DoH mentioning they would take over but not having the budget for it
         5. DoH has limited ability to influence what NGOs do – they have the budget, so they make the decisions
   3. Recommendations or persisting gaps/ issues
      1. Medicines availability is critical
         1. Continued support from ICRC needed
         2. Improvement of medicines distribution needed
         3. Improvement of supply chain planning
      2. Incentives for staff, such as nurses, lab staff, or data collectors
         1. No need for incentives because should be covered by salary
      3. Daily reporting of patient data with description of key data points
      4. Lab investigations should be available
      5. Face-to-face health education for patients
         1. NCD training for health educators
      6. Proactive involvement of authorities and advocacy for appropriate budgets
      7. Better integration and referral system in health system (could us newly established “grid”)
2. **Perceived patient health seeking/ preferences**
   1. Private-sector use if medicines unavailable in PHCC/public facilities
      1. Some patients not being able to afford medicines from the private sector
   2. Preference of Gulan over PHCC
      1. Familiarity with staff (because NCD registration started in 2017)
      2. Gulan is a bigger hospital
      3. Doctors in PHCC are changing frequently and only available few days per week
      4. Shortage of doctors at PHCCs
   3. Lack of access to healthy food options or resources for activities
   4. Patients’ perception
      1. Patients’ (and doctors’) mistrust towards nurses
      2. Patients’ need to feel like they are looked after
   5. Patients' information about NCDs
      1. Sources of information
         1. From relatives and peers
         2. Internet or TV
      2. Know about signs and symptoms, not much knowledge beyond
   6. Patients seek care at tertiary or private facility for diagnosis
3. **General health system descriptions**
   1. Financing and governance
      1. Staff not getting paid for years or only part of their salary
      2. Covid-19 being the health system priority
      3. DoH relying on the Iraqi MoH for financial support (and purchasing medicines)
      4. Budget of MoH not taking population movement into account
   2. Healthcare staff and services
      1. General overburdening of health professionals and them being tired because of covid-19
      2. Screening program with data at district level exists
      3. Big turnover of staff, particularly in rural areas
   3. Medicines and supply chain
      1. Supply chain planning
         1. Yearly estimation of next two years’ medicines need sent to Iraqi MoH
         2. Estimation based on buffer from last year’s consumption (+15-20%)
            1. Usually too small estimations
         3. Push system, not needs based
      2. Lack of inventory system in facilities
   4. Information system
      1. Chronic disease ID and booklet available
         1. Patient carries booklet around (info included)
         2. Renewed after two years at Gulan hospital
      2. Newly introduced grid or network – laptops linked via Viber – after start of decentralisation, not being used for decentralisation – resistance of DOH]]
   5. Destruction/impact on health system of 40 years of war
4. **Community-level interventions**
   1. Structure, Role, Activities of IRCS in general
   2. Role and adaptations of IRCS in relation to Covid
   3. Role of IRCS in relation to NCDs and potential future role.
5. **Cross-cutting themes**
   1. NGOs operational realities
      1. Lack of integration or coordination of NGOs (having specific mandates, not being able to work with all actors or not consistently using chronic disease ID)
      2. Challenges for NGOs to implement projects with stronger engagement and involvement components
      3. Increasing donor and reporting requirements
   2. Effect of covid and restrictions
      1. Closure of facilities
      2. Patients afraid of seeking care
   3. Cultural factors
      1. Role of women in society
   4. Corruption and fraud
   5. Limitations of humanitarian organizations in view of health system needs
