# Supplementary material for: Decentralising healthcare for diabetes and hypertension from secondary to primary level in a humanitarian setting in Kurdistan, Iraq: a qualitative study
Source: BMC Health Serv Res. 2025 Apr 15;25:548. doi: 10.1186/s12913-025-12571-6 (PMC11998334; doi:10.1186/s12913-025-12571-6)
Supplement: Supplementary file 5 — Supplementary Material 5. [file 12913_2025_12571_MOESM5_ESM.docx]

Iraq **Patient** Coding Framework: Initial themes

*These are mainly descriptive themes to group the data from the transcripts. They reflect the main topics that were discussed in the interviews (which is a product of what was suggested in the topic guides, what was asked to patients, and what patients responded to/wanted to talk about). Some will break down further, e.g. into challenges, positive/negative experiences etc. Our interpretation of the data in those groupings will follow as we code. We will then come back to the conceptual framework which guided the topic guide and look at how the domains identified from the data relate to those domains.*

*There are 16 transcripts; for the final 6 the topic guide was adapted to focus more on patients’ experiences of living with their condition, lifestyle (diet/exercise), and specific experiences in relation to decentralisation (not a lot of response re the latter).*

1. *[Added as Nvivo case classification]* Patient description: age, gender, education level, where the patient lives/ has lived, and family description, time since diagnosis, refugee/IDP status
2. Diagnosis

- Prompts for diagnosis
- Process and place of diagnosis location and time
- Reported in relation to life events
- Information after diagnosis
- Process for second diagnosis
- Response to/feelings about diagnosis

1. Local knowledge and awareness of condition

- Content of local knowledge
- Sources of local knowledge or awareness

1. Challenges and strategies of living with condition

- Emotional challenges
- Practical challenges
- Strategies to cope with disease challenges

1. Family/colleagues/friends support for patient

- Emotional support
- Information sharing
- Practical support

1. Experiences at local health centre

- Description of services
  - Available services
  - Not available services
- Medication
  - Availability, strategies when not available
  - Quality of medicines
  - Costs of medicines
- Tests
  - Availability, strategies when not available
  - Costs
- Attitude and demeaner of staff
- Frequency of visits
- Processes for follow up
  - Patient initiated
  - Provider initiated

1. Experiences at Gulan hospital
2. Comparisons between PHCC/Gulan Hospital/other services (places, times, private sector)

- Availability of services and medicines
- Transport/distance/accessibility
- Costs
- Perceived quality (medicines, health professionals, etc.)

1. Private care

- Types of services (x from data)
  - Eye tests/care
- Costs and discontinuation of care or medicines due to it

1. Diet:

- Descriptions of advice received and their sources
- How patients interpret/feel about/follow/don’t follow advice

1. Exercise

- Descriptions of advice received and their sources
- How patients interpret/feel about/follow/don’t follow advice

1. Patient’s suggestions for improvements/changes to services
2. Cross-cutting themes:

- Responsibility for follow-up
- Differences of care in place and time
- Sources of information
- Direct comments on decentralisation
- Covid-19 changes (or lack thereof; effect on livelihood)
- Role of religion

1. Decision-making on care-seeking
